# Labour Trafficking among Men and Boys in the Greater Mekong Subregion: Exploitation, Violence, Occupational Health Risks and Injuries

**DOI:** 10.1371/journal.pone.0168500

**Published:** 2016-12-16

**Authors:** Nicola S. Pocock, Ligia Kiss, Sian Oram, Cathy Zimmerman

**Affiliations:** 1 Gender Violence and Health Centre, Department of Global Health and Development, London School of Hygiene and Tropical Medicine, London, United Kingdom; 2 Section of Women’s Mental Health, Institute of Psychiatry, Kings College London, London, United Kingdom; Simon Fraser University, CANADA

## Abstract

**Background:**

Men comprise nearly two-thirds of trafficked and forced labourers in common low-skilled labour sectors including fishing, agriculture and factory work. Yet, most evidence on human trafficking has focused on women and girls trafficked for sex work, with scant research on trafficked men and boys.

**Methods:**

We analyse survey data from the largest systematic consecutive sample of trafficked people collected to date to describe the prevalence of violence, occupational health risks and injuries and associated factors. Participants were labour-trafficked men and boys using post-trafficking support services in Thailand, Cambodia and Vietnam.

**Findings:**

Data are presented on 446 males aged 10–58. Men and boys were mainly trafficked for fishing (61.7%), manufacturing (19.1%) and begging (5.2%). Fishermen worked extensive hours (mean 18.8 hours/day, SD 5.9) and factory workers worked on average 11.9 hours/day (SD 2.9). 35.5% of male survivors had been injured while trafficked; 29.4% received no personal protective equipment (e.g. gloves). The most commonly reported injuries among all males were deep cuts (61.8%) and skin injuries (36.7%), injuries for which fewer than one-quarter reported receiving medical care. Six fishermen lost body parts, none of whom received medical care. Most males (80.5%) had no or very few rest breaks. One-third (37.8%) experienced severe violence. Work-related injuries were associated with severe violence (AOR 3.44, CI:1.63–7.26), being in the fishing sector, (AOR 4.12, CI:2.39–7.09) and threats (AOR 2.77, CI:1.62–4.75). Experiencing any violence was associated with threats (AOR 26.86, CI:14.0–51.23), being in the fishing sector (AOR 18.53, CI:8.74–39.28) and fluency in language of destination country (AOR 0.39, CI:0.20–0.75).

**Conclusion:**

This study highlights the abuse and extreme occupational hazards suffered by trafficked men and boys. Occupational health and safety interventions are urgently needed to protect male migrant labourers working in high-risk sectors, particularly fishing.

## Introduction

Human trafficking is a crime of extreme exploitation that affects men, women, and children globally. While there is a growing body of literature on the health of female trafficking survivors [1], there is currently little to no evidence on the health needs of trafficked men and boys. To date, few studies have measured violence, occupational risks and injuries experienced by male trafficking survivors, despite the substantial likelihood of injury and enduring health problems in work sectors into which men and boys are commonly trafficked [2,3]. International Labour Organization (ILO) estimates suggest that men comprised 60% of total forced labour in sectors including fishing, agriculture and factory work in 2012 [4].

It is estimated that in the Asia-Pacific region there are 11.7 million forced labourers, who comprise 56% of forced labourers globally [4]. In the Greater Mekong Subregion (GMS), there is rapidly growing recognition of the sectors in which migrant men and boys are commonly exploited, in particular, the long-haul fishing industry [5–7]. Recent media reports have graphically depicted deplorable abuses, imprisonment and other slave-like treatment of men and boys recruited onto fishing vessels [8,9]. This study aimed to explore the prevalence of occupational health risks, injuries and violence and associated factors among men and boys trafficked for fishing, manufacturing and other sectors.

### Human trafficking, migrant workers and health and safety

Migrant workers, especially those labouring in high risk, under-regulated sectors comprise a substantial proportion of victims of labour trafficking [2,10]. Compared to non-migrants, migrant workers in these sectors are often assigned the most dangerous tasks and are likely to experience more hazardous work and living conditions whether they are trafficked or not, often leading to higher rates of morbidity and mortality. Research shows that migrant workers have greater risk of fatal and non-fatal injuries compared to native populations, even when performing the same job [3,11,12]. They frequently receive inadequate safety and occupational training and rarely have specialist skills or previous experience [2]. Limited local language ability can prevent migrant workers from asking questions, expressing concerns about occupational safety and health (OSH) and make it equally difficult for employers to provide training and guidance [2,10]. While personal protective equipment (PPE) should meet industry standards, safety gear is often absent or under-utilized by migrant workers and rarely, if ever, offered to trafficked workers [13]. Furthermore, migrants, particularly irregular migrants, are rarely aware of or willing to assert their rights to safe work conditions. Migrant workers with undocumented status in the USA, for example were generally accepting of unsafe work conditions, extended hours and even concealed injuries, fearing job loss if they complained [3,14,15]. Workers without identity documents are often excluded from compensation and insurance schemes, leaving them unable to seek necessary healthcare and support [14,16,17]. Exiting hazardous situations can be difficult for migrant workers because of debt repayment obligations and because they are often owed back-pay by employers [3,15].

When migrant workers are trafficked, the hazards of these conditions are exacerbated, and often include physical and/or sexual abuse and severe deprivation, making injuries, illness and distress nearly inevitable. A study among male trafficking survivors in the UK found that 42.3% of men reported violence and 32.7% experienced injury during trafficking [18]. The limited research on trafficked men also suggests the links between acts associated with human trafficking and mental health problems, specifically indicating that injuries during trafficking, restricted freedom, physical violence, fear of traffickers and lack of a confidante were associated with symptoms of depression, anxiety and post-traumatic stress disorder [18].

### Workplace violence

According to the ILO, workplace violence involves “any action, incident or behaviour that departs from reasonable conduct in which a person is assaulted, threatened, harmed, injured in the course of, or as a direct result of, his or her work”[19,20].

Research on workplace-related interpersonal violence among migrant male labourers is scarce, with few studies conducted with irregular migrant workers [20]. Among 116 migrant farm workers in the USA, violence was more commonly experienced by migrants than native workers, attributable to poor living conditions, crowding, lack of recreational activities and availability of alcohol [21]. According to the ILO, homicide was the leading cause of workplace death among foreign-born workers in the US [20]. Sexual violence against men and boys in all settings, including the workplace, is significantly under-researched [22]. Among migrant workers in Asia, qualitative reports note physical and sometimes sexual violence perpetrated by police and authorities [23,24]. However, abuse appears to be poorly documented, perhaps because of workplace sub-cultures where even severe physical assaults might be normalized or ignored [20,25].

Violence, threats and imprisonment are common in trafficking situations. One in ten males in a survey of 596 commercial fishermen in Thailand was beaten severely [26]. Trafficked fishermen have reported being abused when they complained, were found resting, were not working fast enough or did not understand instructions [27–29]. Being too sick or weak to work has also led to beatings or even murder by boat captains [5].

### Work hazards

Work-related hazards are common in jobs done by migrant labourers, especially men and boys trafficked into particularly dangerous industries such as commercial fishing, manufacturing, agriculture and construction [3]. In the GMS, increasing numbers of so-called ‘sea-slaves’ are being identified in Southeast Asian waters [8,30]. Commercial fishing is among the world’s most dangerous occupations [31], involving 24-hour work cycles of physically demanding tasks, poor use of protective gear, crew inexperience and high injury rates, from for example, deep cuts from fishing equipment, falls on deck or into the sea and drowning [32–34]. Adverse weather and night working also increase accident risk [35,36]. While commercial fishing generally demands long work shifts, trafficked fishermen tend to work for even more extensive periods, often labouring for weeks or months at a time without respite [5,26–28]. Despite the high risk of injury and illness, most reports on migrant and trafficked fishermen note limited or no access to healthcare [27,37]. In a study comparing migrants’ working conditions across multiple sectors in Thailand, higher proportions of fishermen experienced poor working conditions, including document confiscation, delayed payments and inadequate rest compared to factory or agricultural workers [38].

Industrial or factory work for electronics and consumer goods also employ high numbers of migrant workers, a proportion of whom work in forced labour conditions and are exposed to work-place hazards including harmful chemicals, airborne particles, machine-related injuries and extreme time pressures [39]. A study in China showed that migrant workers regularly disregarded safety precautions to increase work speed [39]. Factory workers report suffering physical assaults by management, machine-related amputations, crushed body parts, fractures and musculoskeletal pain [40–42]. Research shows that trafficked factory workers are rarely given adequate, if any, protective gear, have very restricted movement and are often subjected to violence [43–47].

Occupational hazards are similar for agricultural workers, also a common sector for trafficked labour, including injuries from heavy farm machinery and chemical hazards that cause skin and respiratory diseases [48,49]. Studies have shown that risk factors for injuries among agricultural workers were being male; having an education level beyond high school; having fewer than five years of machine operating experience; daytime drowsiness; being in debt; having lower family income; and feeling stressed [49,50].

Risks associated with construction work include: exposure to harmful chemicals; lifting heavy objects or repetitive motions; overexposure to UV rays; falls from working at heights; crush injuries; being struck by falling objects and injuries from hand tools [51]. Among male construction workers in China, leading causes of injuries included collisions, cuts/piercings and falls [52]. Injuries were significantly associated with heavy smoking; serious alcohol consumption; no injury prevention and safety education; and depressive symptoms [52]. This study is the first to explore occupational health risks and injuries amongst labour trafficked men and boys in these hazardous industries.

## Methods

### Study design and participants

This study analyses data from a first interview with 446 males who participated in the Study on Trafficking, Exploitation and Abuse in the Mekong (STEAM) and who reached the country of exploitation. All males were trafficked across borders except 11 boys aged 12–17 who were trafficked internally, mainly for begging in Thailand (n = 9). STEAM is a multi-site, longitudinal survey carried out with men, women and children receiving post-trafficking assistance in Thailand, Cambodia and Vietnam. A two stage sampling strategy was employed. First, the study team selected fifteen post-trafficking service providers in Thailand, Cambodia and Vietnam based on diversity of clientele (age, sex, sector of exploitation, country of origin), relationship with country teams at the International Organization for Migration (IOM) and agreements with government agencies [53]. Second, a consecutive sample of individuals participated in face to face interviews within 0–14 days of entry to the service, and 30–90 days later if they were still in contact with services [53,54]. Males were identified and interviewed in 7 of the 15 services at first interview. Service providers received clients referred from police and immigration services, non-governmental and international organizations and government agencies (e.g. Cambodia’s Department of Anti-Trafficking and Juvenile Protection, Thailand’s Department of Social Development and Welfare, Vietnam’s Department of Social Evils Prevention) [53,54]. These countries may have different legal definitions of human trafficking that influence differing screening criteria used to identify trafficked persons. For example, potential victims in Thailand and Cambodia undergo an initial screening process by first responders. The Cambodian assessment form applies debt bondage as a trafficking indicator, whereas the Thai form does not [55,56], as “debt bondage” is not included in Thailand’s current legislative definition of human trafficking [57,58]. Criteria used to determine who is eligible for services also varies. For example, in Thailand, data represented individuals using government-operated services who had agreed to participate in legal cases against traffickers [59]. In Vietnam, data are from individuals using government-contracted services. Only Vietnamese citizens can be referred to these services. The STEAM study describes individuals who received post-trafficking services, regardless of differing legal definitions of trafficking and service eligibility criteria between countries [53].

### Data collection

The survey instrument was translated into Khmer, Thai, Vietnamese, Burmese, and Lao, refined through group discussions with IOM counter-trafficking teams, further revised through pilot-testing, and reviewed after back-translation into English [53]. Interviews were conducted onsite at services by social workers or caseworkers, following intensive training by the principal investigator (LK) and the IOM, with interpreters when needed. Data collection and entry were coordinated by the local IOM offices, with oversight by the London School of Hygiene and Tropical Medicine between October 2011 and May 2013 [53].

### Ethics

Interviewers were recruited from participating shelter staff, selected social workers (Thailand) and International Organization for Migration partners, and were trained to follow a strict ethics protocol based on the World Health Organization Ethical Recommendations for Interviewing Trafficked Women (authored by one of the STEAM Principle Investigators) [60]. Guidance included ensuring participation was voluntary and confidential, avoiding and managing distress, e.g. by asking questions in non-judgmental ways and affirming responses in a supportive manner, alongside outlining options for supported referral. Survey participants were identified and interviewed by experienced service providers, and for those who were under 18 years old, the child’s care team were consulted. All interviewers had the professional training or experience to identify when an individual should not be interviewed, when to stop or pause an interview, how to respond to distress and when to make necessary referrals. During consent procedures, the interviewer explained the study content and option to refuse or interrupt participation without consequences for services provision [61]. The response rate among men and boys was 100%, although one male did not finish the interview and was referred for health services. Written consent was given by name or thumbprint among adults and children, with additional written consent by a legal guardian for children aged under 18. Data were anonymized and questionnaires were stored securely in each country. Ethical approval was obtained from the London School of Hygiene and Tropical Medicine and national ethics boards in each of the study countries [54].

### Variables

The survey instrument was adapted from a prior European study on health and trafficking, and comprised sections on socioeconomic background, pre-trafficking experiences, living and working conditions during trafficking, violence, health and post-trafficking emotional wellbeing and concerns [62].

#### Violence

Questions on abuse exposures were developed from the violence and health outcome modules of the WHO multi-country study on women’s health and domestic violence [63]. A categorical outcome variable for violence was calculated based on the categories often used in violence studies, with the following classified as “severe violence”: being kicked, dragged, beaten up, tied or chained, choked or burned; having a dog released to bite or scratch, being threatened with a weapon, cut with a knife, shot and forced to have sex. “Less severe violence” was classified as: slaps, pushes and hits [53,64].

#### Injury and occupational hazards

A binary variable for “ever having experienced a work-related injury” was created. This outcome variable included injuries that were incurred at work but not related to violence; the variable “injuries from violence” had a high proportion of missing data and is not included in this analysis. The occupational health risks (OHR) score was created by combining binary variables on occupational hazards and presence of personal protective equipment (PPE) for that hazard. For example, “long hours in the sun without a break” and “sun hat” would give a binary score of zero OHR, whereas “long hours in the sun without a break” and “no sun hat” would give a score of 0.5 (conservative approach assuming the hat is 50% protective) for this OHR. OHRs were then summed per sector and the proportion endorsed by each sector was calculated, with minimum score 0 and maximum 100. The OHR score was entered as a continuous variable in bivariable and multivariable models.

#### Working and living conditions

A categorical variable for hours worked/day was created as was a variable for days worked/week. A continuous variable for living conditions was created summing the number of items endorsed for living conditions; a higher score indicates poorer living conditions. Binary variables for sector (fishing or other), weekly rest day, cheated of wages and no documents (either had none or had documents but these were withheld) were created, as was a continuous variable for wages received/day. A binary variable was created for restricted freedom, depending on whether a participant endorsed the items “Ever been locked in a room” and/or was “Never” free [53].

#### Language proficiency

A binary variable “fluent” for whether participants spoke the language of destination country was also created. While many trafficked fishermen were repatriated from Indonesia and third countries, correspondence with IOM indicates that almost all of these men and boys were trafficked via Thailand on Thai owned boats [65]. The fluent variable was therefore coded among fishing sector males as positive if the participant spoke Thai and the destination country was Malaysia, Indonesia, Mauritius or Thailand [65].

### Data analysis

**Fig 1:**
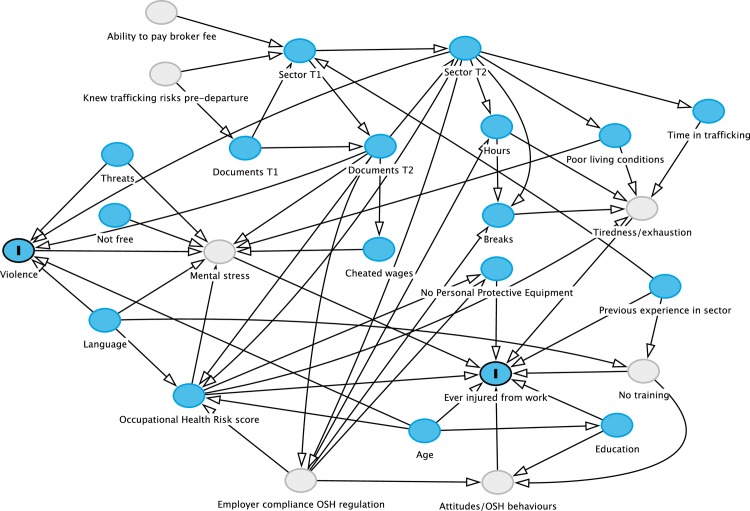
Directed Acyclic Graph of effect of exposures on work related injuries and violence among trafficked males.

As our analysis is exploratory, we drew Directed Acyclic Graphs (DAG), mapping our assumptions and hypotheses conceived a priori (Fig 1). Fig 1 shows the overall conceptual thinking behind the analyses–a complete list of DAGs can be found in S1 Fig. DAGs were used to identify exposures for which we could estimate associations with the outcomes with minimal bias and thus include as covariates in statistical models. Initial selection of covariates was based on literature regarding the causes of injuries among migrant and trafficked workers. Variables hypothesized to cause injuries were hours and OHR score (primary exposures), cheated wages, violence and threats (secondary exposures) and language and documents (tertiary exposures). Further explanation can be found in S1 File.

Bivariable logistic regression was used to describe the frequency and distribution of exposure variables with each outcome variable (work-related injuries or any violence experienced). Adjusted odds ratios were then calculated for the exposure variables to describe their relationship with the outcomes using multivariable logistic regression. Age and time in trafficking were included as continuous variables in multivariable models to avoid loss of information [66]. A quadratic term for age and a cubic term for months in trafficking, were created and included in multivariable models, based on their respective theorized relationships to injuries and/or violence (conceived after graphically examining bivariable scatterplots with lowess smoothing lines) [67,68]. The age variable was centered on the mean, and months in trafficking centered on the median to aid interpretation in models. All theorized primary, secondary and tertiary exposures for injuries were included in multivariable models, not solely those that were statistically significant in bivariable analyses [69].

The effects of the primary exposures (hours and OHR score) on injuries could not be estimated because the variable “Employer compliance with OSH regulation” was unobserved and could not be controlled for, violating the assumptions of the DAG; for further explanation, please see S1 File and S1 Fig. We present the unadjusted and adjusted odds ratios for hours and OHR score as indicative of possible relationships; however they must be interpreted with caution and not as indicating causality. Analyses were conducted in Stata 14 [70].

## Results

### Participant characteristics

The majority (71.6%) of the 446 males in this study were aged 18–34 and most were trafficked for fishing (n = 275, 61.7%) and factory work (n = 85, 19.1%) (Table 1). Males in other industries included child beggars (n = 23), men and boys trafficked for agriculture (n = 21) and construction work (n = 16) and small-scale industries like car care (n = 5) and home businesses (n = 5). Fishermen were primarily from Cambodia (78.9%) and most factory workers (80.0%) were from Vietnam. Education levels differed by sector, with 22.9% of fishermen not having any formal education compared to 2.4% among factory workers and 14.0% among workers in other sectors. The main destination countries were Indonesia (46.9%) for fishermen and China (80.0%) for factory workers. Approximately half of participants in other sectors, including agriculture and begging (55.8%), worked in Thailand. Over one third (38.9%) of fishermen spent more than two years in the trafficking situation, whereas the majority of factory workers (61.2%) were trafficked for between 1 and 6 months.

**Table 1:** Characteristics of labour-trafficked men and boys accessing post-trafficking services in Thailand, Cambodia and Vietnam (n = 446).

	Whole sample (n = 446)		Fishing (n = 275)		Factories (n = 85)		Other (n = 86)	
	N	%	N	%	N	%	N	%
**Age**								
10 to 14	27	6.1%	1	0.4%	-	-	26	30.2%
15 to 17	36	8.1%	12	4.4%	9	10.6%	15	17.4%
18 to 24	168	37.7%	103	37.5%	47	55.3%	18	20.9%
25 to 34	151	33.9%	123	44.7%	14	16.5%	14	16.3%
> = 35	64	14.3%	36	13.1%	15	17.6%	13	15.1%
**Mean age (SD) (range)**	446	25.6 (8.1) (10–58)	275	27.0 (6.9) (12–55)	85	24.9 (8.1) (15–55)	86	21.9 (10.1) (10–58)
**Education**								
Primary or less (1–5 grade)	217	48.7%	136	49.5%	29	34.1%	52	60.5%
Secondary (6–8 grade)	125	28.0%	63	22.9%	43	50.6%	19	22.1%
Higher (10–11 grade)	22	4.9%	10	3.6%	11	12.9%	1	1.2%
University degree	3	0.7%	3	1.1%	-	-	-	-
No formal education	77	17.3%	63	22.9%	2	2.4%	12	14.0%
Missing data	2	0.4%	-	-	0	-	2	2.3%
**Country of origin**								
Cambodia	257	57.6%	217	78.9%	12	14.1%	28	32.6%
Laos	5	1.1%	-	-	-	-	5	5.8%
Myanmar	65	14.6%	55	20.0%	4	4.7%	6	7.0%
Thailand	12	2.7%	2	0.7%	1	1.2%	9	10.5%
Vietnam	106	23.8%	-	-	68	80.0%	38	44.2%
Other	1	0.2%	1	0.4%	-	-	-	-
**Country of destination**								
China	107	24.0%	2	0.7%	68	80.0%	37	43.0%
Malaysia	30	6.7%	28	10.2%	2	2.4%	-	-
Thailand	140	31.4%	77	28.0%	15	17.6%	48	55.8%
Vietnam	1	0.2%	-	-	-	-	1	1.2%
Indonesia	129	28.9%	129	46.9%	-	-	-	-
Mauritius	33	7.4%	33	12.0%	-	-	-	-
South Africa	6	1.3%	6	2.2%	-	-	-	-
**Speaks language of destination country**	142	31.8%	113	41.1%	2	2.4%	27	31.4%
**Sector of exploitation**								
Sex workers	1	0.2%	-	-	-	-	1	1.2%
Animal farming/meat preparation	3	0.7%	-	-	-	-	3	3.5%
Agriculture/farming/plantation	21	4.7%	-	-	-	-	21	24.4%
Begging	23	5.2%	-	-	-	-	23	26.7%
Car care	5	1.1%	-	-	-	-	5	5.8%
Domestic worker/cleaner	2	0.4%	-	-	-	-	2	2.3%
Construction	16	3.6%	-	-	-	-	16	18.6%
Factory	85	19.1%	-	-	-	-	-	-
Fishing	275	61.7%	-	-	-	-	-	-
Home business	5	1.1%	-	-	-	-	5	5.8%
Street seller/shop	5	1.1%	-	-	-	-	5	5.8%
Other	5	1.1%	-	-	-	-	5	5.8%
**Has previous experience in sector**	38	8.5%	18	6.6%	4	4.7%	16	18.6%
**Time in trafficking (months)**								
<1	41	9.2%	8	2.9%	9	10.6%	24	27.9%
1 to 6	156	35.0%	58	21.1%	52	61.2%	46	53.5%
7 to 12	51	11.4%	36	13.1%	10	11.8%	5	5.8%
13 to 23	67	15.0%	62	22.5%	5	5.9%	-	-
> = 24	110	24.7%	107	38.9%	-	-	3	3.5%
Missing data	21	4.7%	4	1.5%	9	10.6%	8	9.3%
**Median months in trafficking (median absolute deviation)**	425	6.3 (5.5)	271	16.0 (11.5)	76	3.0 (1.0)	78	1.9 (0.0)
**Country of service access**								
Thailand	105	23.5%	56	20.4%	13	15.3%	36	41.8%
Cambodia	235	52.7%	219	79.6%	4	4.7%	12	14.0%
Vietnam	106	23.8%	-	-	68	80.0%	44	44.2%

### Occupational hazards and exploitation

The most common occupational health risks reported by all males were repeated bending or lifting (75.6%), and lifting heavy objects (69.9%) (Table 2). Almost one-third of participants (29.4%) reported having been given no personal protective equipment. Nearly all fishermen (96.7%) reported working long hours in the sun, cold or wet without a break. Fishermen worked the longest hours per week (mean of 132.3 hours, SD 40.2), with over a third (41.8%), labouring 20 or more hours per day. Factory workers worked approximately half the hours of fishermen (mean of 79.6 hours/week, SD 21.3). The vast majority of trafficked males (86.5%) worked everyday and 80.5% had no or very few rest breaks.

**Table 2:** Occupational hazards, exploitation and violence during trafficking among men and boys using post-trafficking services in Thailand, Cambodia and Vietnam (n = 446).

	Whole sample (n = 446)		Fishing (n = 275)		Factories (n = 85)		Other (n = 86)	
	N	%	N	%	N	%	N	%
**Occupational hazard**								
Repeated bending or lifting	296/408	75.6%	261/275	94.9%	15/85	17.7%	20/48	41.7%
Lift heavy objects	285/408	69.9%	257/275	93.5%	12 of 85	14.1%	16/48	33.3%
Use sharp instruments	179/408	43.9%	154/275	56.0%	12 of 85	14.1%	13/48	27.1%
Work with harsh chemicals, cleaning solutions	91/408	22.3%	52/275	18.9%	32/85	37.7%	7/48	14.6%
Working in dusts or fibers	28/106	26.4%	-	-	22/85	25.9%	6/21	28.6%
Operating big or heavy machinery	21/101	20.8%	-	-	19/85	22.4%	2/16	12.5%
Working up high off the ground	13/101	12.9%	-	-	3 of 85	3.5%	10/16	62.5%
Working with raw meat	2 of 24	8.3%	-	-	-	-	2/24	8.3%
Working with or near pesticides	4 of 24	16.7%	-	-	-	-	4/24	16.7%
Unstable or heavy work platforms	226/275	82.2%	226/275	82.2%	-	-	-	-
Work along rocky coasts or in remote offshore	179/275	65.1%	179/275	65.1%	-	-	-	-
Small, unstable or badly maintained fishing vessel	94/275	34.2%	94/275	34.2%	-	-	-	-
Badly maintained or no fishing equipment	77/275	28.0%	77/275	28.0%	-	-	-	-
No safety/bad or no survival equipment	170/275	61.8%	170/275	61.8%	-	-	-	-
Long hours in the sun, cold or wet without a break	266/275	96.7%	266/275	96.7%	-	-	-	-
Working near road traffic	26/57	45.6%	-	-	-	-	26/57	45.6%
Long hours in the sun without a break	30/57	52.6%	-	-	-	-	30/57	52.6%
Long hours in the cold or wet without a break	19/57	33.3%	-	-	-	-	19/57	33.3%
**No personal protective equipment given**	131	29.4%	37	13.5%	33	38.8%	61	70.9%
**Mean OHR score (SD)&**	441	39.3 (23.3)	275	49.5 (17.5)	85	16.1 (16.9)	81	28.7 (24.5)
**Hours worked per day**								
< = 8	40	9.0%	15	5.5%	1	1.2%	24	27.9%
8 to 10	44	9.9%	5	1.8%	22	25.9%	17	19.8%
11 to 15	57	12.8%	25	9.1%	18	21.2%	14	16.3%
16 to 19	23	5.2%	21	7.6%	2	2.4%	-	-
> = 20	117	26.2%	115	41.8%	2	2.4%	-	-
No fixed hours	161	36.1%	94	34.2%	39	45.9%	28	32.6%
Don't know	4	0.9%	-	-	1	1.2%	3	3.5%
Hours worked per day mean (SD)*	281	15.6 (6.8)	181	18.8 (5.9)	45	11.9 (2.9)	55	8.2 (3.6)
**Days worked/week**								
6 or fewer	24/443	5.4%	4/274	1.5%	11 of 83	13.3%	9/86	10.5%
Everyday	383/443	86.5%	267/274	97.5%	63 of 83	75.9%	53/86	61.6%
No fixed days	36/443	8.1%	3/274	1.1%	9 of 83	10.8%	24/86	27.9%
Hours worked/week mean (SD)*	280	108.9 (47.7)	180	132.3 (40.2)	45	79.6 (21.3)	55	56.3 (25.8)
**Could not change hours/time off if sick/holiday**	367	82.3%	239	86.9%	74	87.1%	54	62.8%
**Cheated of wages**	314	70.4%	195	70.9%	71	83.5%	48	55.8%
**Median wage (USD/day) (median absolute deviation)**	128	3.5 (2.8)	79	1.4 (1.1)	13	9.5 (0)	36	6.3 (3.2)
**No or very few rest breaks**	359	80.5%	245	89.1%	67	78.8%	47	54.7%
**At least one hazardous living condition**	430	96.4%	275	100.0%	85	94.1%	75	87.2%
**Living situation mean score (SD)&&**	446	4.2 (2.5)	275	5.6 (1.8)	85	1.7 (1.4)	86	2.2 (1.8)
**Threats to self or loved ones**	254	57.0%	191	69.5%	31	36.5%	32	37.2%
**No documents**	369	83.1%	208	75.9%	79	92.9%	82	96.5%
**Restricted freedom**	323	72.4%	232	84.4%	49	57.7%	42	48.8%
Violence severity**								
No violence	219	49.3%	79	28.7%	72	86.8%	68	79.1%
Less severe violence	57	12.8%	48	17.5%	2	2.4%	7	8.1%
More severe violence	168	37.8%	148	53.8%	9	10.8%	11	12.8%
**Violence type**								
Physical violence^^	215	48.2%	188	68.4%	10	11.8%	17	19.8%
Sexual violence^	6	1.4%	5	1.8%	-	-	1	1.2%
Physical or sexual violence, or both**^^	215	48.4%	188	68.4%	10	12.1%	17	19.8%
**Violence exposures (selected)**								
Slapped you, shoved you or threw something that could hurt you	163	36.6%	145	52.7%	5	5.9%	13	15.1%
Pushed or shoved you	159	35.6%	142	51.6%	7	8.2%	10	11.6%
Hit with a fist or something that could hurt you	143	32.1%	123	44.7%	9	10.6%	11	12.8%
Kicked, dragged or beat you up	112	25.1%	101	36.7%	5	5.9%	6	7.0%
Threatened to use gun, knife or weapon against you	103	23.1%	91	33.1%	5	5.9%	7	8.1%
**Violence perpetrator**								
Employer (when not agent, trafficker)	109/225	48.4%	92/196	46.9%	4 of 11	36.4%	13/18	72.2%
Trafficker	45/225	20.0%	38/196	19.4%	4 of 11	36.4%	3/18	16.7%
Co-worker	51/225	22.7%	47/196	24.0%	2 of 11	18.2%	2/18	11.1%
Police/authority/government official	9/225	4.0%	9/196	4.6%	-	-	-	-
Family member or acquaintance	9/225	4.0%	5/196	2.6%	1 of 11	9.1%	3/18	16.7%
Security staff/bouncer	57/225	25.3%	55/196	28.1%	-	-	2/18	11.1%
Other	8/225	3.6%	7/196	3.6%	-	-	1/18	5.6%

&Min score 0, max 100

&&Min score 0, max 9

*Among n = 280 males who worked fixed hours/could recall them

**2 missing whole sample

^3 refused to answer

^^excluding being threatened with a weapon

Almost all males experienced at least one bad living condition (96.4%), on average experiencing four (SD 2.5) bad living conditions. Three-quarters of fishermen (75.9%) reported having no documents or having their papers kept from them, compared to almost all factory workers (92.9%) and workers in other sectors (96.5%). Nearly three-quarters of survivors (72.4%) reported severely restricted freedom, i.e., “never” being free or being locked in a room. Most (70.4%) reported being cheated of wages.

### Violence

Nearly one in two survivors (48.2%) were subjected to physical violence and 37.8% reported severe violence, including 23.1% who were threatened with a weapon and six males who suffered sexual violence. Half of the fishermen (53.8%) experienced severe violence compared to approximately one in ten factory and other workers (10.8%; 12.8%). The main perpetrators of reported violence were employers (48.4%), security staff/bouncers (25.3%) and co-workers (22.7%). For fishermen, perpetrators were often the boat foremen. Threats were common, especially among fishermen; twice the proportion of fishermen (69.5%) reported threats to self or loved ones compared to 36.5% of factory workers and 37.2% of other workers.

### Injuries and healthcare access

One-third (35.5%) of men and boys had been injured at least once, of whom 47.0% reported still experiencing pain or difficulty from their injuries at the time of interview (Table 3). Deep or long cuts were the most commonly reported injury (61.8%), followed by skin damage or injury (36.7%), which was particularly common among factory workers (72.7%). Back or neck injuries were sustained by 35.3% of participants, especially among those in sectors other than fishing or factory work (41.7%), such as agriculture and begging. Almost half (45.2%) of those who were ever injured or reported needing healthcare while trafficked did not receive medical care, including for very serious injuries. None of the six fishermen who lost body parts received medical attention. Of 18.0% who sustained a serious head injury, 81.5% did not receive medical attention. Of those reporting an injury or that they needed care while trafficked, 52.3% of fishermen compared to 27.6% of factory workers and 23.3% of other workers, received no care. Of those who did receive care, there was variation in the provider of this care. Higher proportions of factory workers (27.6%) and other workers (23.3%) saw a doctor or nurse compared to only 7.9% of fishermen. One-third (36.5%) of fishermen reported receiving some form of care from traffickers or employers compared to 20.0% of other workers and 6.9% of factory workers.

**Table 3:** Work related injuries among trafficked men and boys using post-trafficking services in Thailand, Cambodia and Vietnam (n = 446).

	Whole sample (n = 446)				Fishing (n = 275)				Factories (n = 85)				Other (n = 86)			
	Injury prevalence (n = 152)		No medical care received		Injury prevalence (n = 128)		No medical care received		Injury prevalence (n = 11)		No medical care received		Injury prevalence (n = 13)		No medical care received	
Injury from work	N	%	N	%	N	%	N	%	N	%	N	%	N	%	N	%
Deep or very long cut	94/152	61.8%	71/94	75.5%	87/128	68.0%	69/87	79.3%	2/11	18.2%	-	-	5/13	38.5%	2/5	40.0%
Very bad burn (not sunburn)	24/150	16.0%	23/24	95.8%	24/127	18.9%	23/24	95.8%	-	-	-	-	-	-	-	-
Serious head injury	27/150	18.0%	22/27	81.5%	26/127	20.5%	22/26	84.6%	-	-	-	-	1/12	8.3%	-	-
Back or neck injury	53/150	35.3%	48/53	90.6%	46/127	36.2%	42/46	91.3%	2/11	18.2%	2/2	100.0%	5/12	41.7%	4/5	80.0%
Skin damage or injury	55/150	36.7%	47/55	85.5%	41/127	32.3%	38/41	92.7%	8/11	72.7%	6/8	75.0%	6/12	50.0%	3/6	50.0%
Broken bone	7/150	4.7%	4/7	57.1%	6/127	4.7%	4 of 6	66.7%	-	-	-	-	1/12	8.3%	-	-
Lost a body part	6/150	4.0%	6/6	100.0%	6/127	4.7%	6 of 6	100.0%	-	-	-	-	-	-	-	-
Eye injury/damage	16/150	10.7%	13/15**	86.7%	15/127	11.8%	12/14	85.7%	1/11	9.1%	1/1	100.0%	-	-	-	-
Ear damage	17/150	11.3%	17/17	100.0%	16/127	12.6%	16/16	100.0%	1/11	9.1%	1/1	100.0%	-	-	-	-
Other^	22/149	14.8%	18/22	81.8%	19/126	15.1%	17/19	89.5%	1/11	9.1%	-	-	2/12	16.7%	1/2	50.0%
**Injury frequency/severity**																
Injured at least once*	152/428	35.5%	-	-	128/273	46.6%	-	-	11/76	14.5%	-	-	13/79	16.5%	-	-
Injured once	66/446	14.8%	-	-	54/275	19.6%	-	-	6/85	7.1%	-	-	6/86	7.0%	-	-
Injured a few times	50/446	11.2%	-	-	41/275	14.9%	-	-	5/85	5.9%	-	-	4/86	4.7%	-	-
Injured many times	36/446	8.1%	-	-	33/275	12.0%	-	-	-	-	-	-	3/86	3.5%	-	-
No injury	276/446	61.9%	-	-	145/275	52.7%	-	-	65/85	76.5%	-	-	66/86	76.7%	-	-
Can't remember	18/446	4.0%	-	-	2/275	0.7%	-	-	9/85	10.6%	-	-	7/86	8.1%	-	-
**Injuries still cause pain/difficulty&**	71/151	47.0%	-	-	66/128	51.6%	-	-	-	-	-	-	5/13	38.5%	-	-
**Healthcare access**																
Ever needed healthcare or was injured from work	237	53.1%	-	-	178	64.7%	-	-	29	34.1%	-	-	30	34.9%	-	-
Received care from doctor or nurse	29/237	12.2%	-	-	14/178	7.9%	-	-	8/29	27.6%	-	-	7/30	23.3%	-	-
Received care from trafficker or employer	72/237	30.4%	-	-	65/178	36.5%	-	-	2/29	6.9%	-	-	6/30	20.0%	-	-
Received care from coworker	27/237	11.4%	-	-	13/178	7.3%	-	-	5/29	17.2%	-	-	9/30	30.0%	-	-
Received care from traditional healer/other	17/237	7.2%	-	-	4/178	2.3%	-	-	6/29	20.7%	-	-	7/30	23.3%	-	-
Received no care	107/237	45.2%	-	-	93/178	52.3%	-	-	8/29	27.6%	-	-	7/30	23.3%	-	-

^3 missing whole sample

&1 missing whole sample

**1 missing from prevalence and medical care

*Excludes those who cannot remember

### Factors associated with injuries and violence

In bivariable analyses, longer hours worked daily was associated with injuries, e.g. working 20 or more hours a day (Unadjusted Odds Ratio 7.66, CI: 2.80–20.97), as was working everyday (UOR 4.40, CI: 1.28–15.02). Other factors associated with injuries in bivariable analyses that were not primary, secondary or tertiary exposures included poor living conditions (UOR 1.45, CI: 1.32–1.60), having no breaks (UOR 2.15, CI: 1.23–3.75) and restricted freedom (UOR 1.88, CI: 1.18–3.01). Men with documents had three times the odds (1/0.33) of experiencing violence compared to men without documents (UOR 0.33, CI: 0.19–0.58).

Multivariable models and their corresponding Directed Acyclic Graphs with a single exposure and outcome are listed in S1 Fig and Table 4. In multivariable analyses, injuries were significantly associated with severe violence (Adjusted Odds Ratio 3.44, CI:1.63–7.26), being in the fishing sector (AOR 4.12, CI:2.39–7.09) and experiencing threats (AOR 2.77, CI:1.62–4.75) (Table 4). Men who received their wages had twice the odds (1/0.42) of being injured compared to those who were cheated of wages (AOR 0.42, CI:0.24–0.71). Men with documents had slightly greater odds (1/0.63) of being injured compared to men without documents (AOR 0.63, CI:0.37–1.07), suggesting a marginal relationship between documents and injuries. The effect of hours worked on injuries may plateau at 16–19 hours (AOR 4.22, CI:1.11–15.99), although we urge caution in interpreting this result (please see limitations section in Discussion).

**Table 4:** Factors associated with work related injuries and any violence experiences among trafficked males using post-trafficking services in Thailand, Cambodia and Vietnam (n = 446).

	Work-related injuries			Any violence		
	Unadjusted Odds Ratio (95% CI)	Adjusted Odds Ratio (95% CI)	Model	Unadjusted Odds Ratio (95% CI)	Adjusted Odds Ratio (95% CI)	Model
**PRIMARY EXPOSURES**						
Hours worked/day			A			
8 to 10	1.75 (0.51–5.93)	1.85 (0.44–7.66)				
11 to 15	3.91 (1.30–11.70)	4.28 (1.31–14.00)				
16 to 19	7.41 (2.13–25.76)	4.22 (1.11–15.99)				
> = 20	7.66 (2.80–20.97)	3.75 (1.25–11.22)				
No fixed hours	2.86 (1.05–7.80)	2.33 (0.78–6.92)				
Days worked/week			A			
Every day	4.40 (1.28–15.02)	2.09 (0.41–10.65)				
No fixed days	1.12 (0.22–5.57)	1.86 (0.22–15.57)				
OHR score	1.02 (1.01–1.03)	1.02 (1.01–1.03)	A			
**SECONDARY EXPOSURES**						
Cheated of wages	0.68 (0.45–1.05)	0.42 (0.24–0.71)	B			
Violence			C			
Less severe	3.21 (1.67–6.16)	1.92 (0.83–4.45)				
More severe	6.72 (4.16–10.85)	3.44 (1.63–7.26)				
Threats	4.80 (3.04–7.58)	2.77 (1.62–4.75)	D	18.76 (11.46–30.68)	26.86 (14.0–51.23)	L
Fishing sector	4.81 (2.93–7.91)	4.12 (2.39–7.09)	E	11.97 (7.42–19.31)	18.53 (8.74–39.28)	L
**TERTIARY EXPOSURES**						
Not fluent in language of destination	0.50 (0.33–0.77)	0.72 (0.42–1.22)	F	0.26 (0.17–0.41)	0.39 (0.20–0.75)	L
No documents	0.43 (0.26–0.72)	0.63 (0.37–1.07)	G	0.33 (0.19–0.58)	0.81 (0.35–1.84)	L
**COVARIATES**						
Age^	1.03 (1.00–1.07)	0.99 (0.95–1.03)	H#	1.01 (0.98–1.04)	0.90 (0.86–0.95)	L
Age^2^	0.99 (0.99–0.99)	0.99 (0.95–1.00)		0.99 (0.99–0.99)	1.00 (0.99–1.00)	L
Months in trafficking^^	1.04 (1.02–1.05)	1.03 (1.01–1.05)	I#			
Months in trafficking^3^	0.99 (0.99–0.99)	0.99 (0.99–1.00)				
Poor living conditions score	1.45 (1.32–1.60)	1.33 (1.14–1.54)	J#			
No previous experience in sector	1.53 (0.72–3.27)	1.60 (0.75–3.44)	K			
No breaks	2.15 (1.23–3.75)					
No PPE	0.51 (0.32–0.82)					
No freedom	1.88 (1.18–3.01)					
Actual wages/day (USD)	0.96 (0.90–1.02)					
Education						
No formal schooling	1.33 (0.74–2.38)					
Primary	1.29 (0.82–2.03)					

Reference group for hours/day: <8 hours, for days/week: 6 or fewer days, for violence: no violence, for education: secondary or higher. Multivariable models (based on DAGs in S1 Fig, explanation in S1 File) included the following: A. Hours (exposure), daysweek, ohr (exposure), age, age^2^, months, months^3,^ B. Cheated wages (exposure), documents, hours, daysweek, ohr, age, age^2^, months, months^3,^ C. Violence (exposure), documents, language, sector, threats, hours, daysweek, ohr, age, age^2^, months, months^3,^ D. Threats (exposure), hours, daysweek, ohr, age, age^2^, months, months^3,^ E. Sector (exposure), documents, previous experience, age, age^2,^ F. Language (exposure), hours, daysweek, age, age^2^, months, months^3,^ G. Documents (exposure), sector, previous experience, age, age^2^, H. Age (exposure), age^2^, months, months^3^, hours, daysweek, I. Months (exposure), months^3^, sector, age, age^2^, hours, daysweek, ohr, J. Living conditions (exposure), ohr, sector, age, age^2^, hours, daysweek, months, months^3,^ K. Previous experience (exposure), age, age^2^, L. Threats (exposure), sector (exposure), language (exposure), documents (exposure), age, age^2^

^Centred at mean (including in quadratic term)

^^Centred at median (including in cubic term)

#This multivariable model was underpowered to detect this effect size, estimate should be interpreted cautiously

Factors significantly associated with violence in the multivariable analysis included threats (AOR 26.86, CI:14.0–51.23), being in the fishing sector (AOR 18.53, CI:8.74–39.28), and language fluency; those who were fluent had twice the odds (1/0.39) of experiencing violence compared to those who were not fluent (AOR 0.39, CI:0.20–0.75). Violence was also slightly associated with younger age (AOR 0.90, CI:0.86–0.95).

## Discussion

In the face of increasing global labour migration, there has been growing recognition that a substantial proportion of individuals will end up in highly exploitative, violent and sometimes fatal circumstances. As the largest study to date on the health of male trafficking survivors, these findings confirm that human trafficking is not limited to women and girls, but that men and boys experience extreme forms of exploitation. Moreover, these abuses of trafficked males are not isolated to a single country or a certain sector, as the men and boys in the study were trafficked to seven countries and into a dozen different labour sectors.

Our findings also confirmed some of the media’s reports of horrors at sea by offering data showing that men and boys who are trafficked for commercial fishing suffer egregious abuses, are exposed to inhuman work hours, and sustain serious injuries, including lost limbs. The results suggest that the unsustainable work hours over months, sometimes years, led to fatigue, accidents, and increased risk of injury. While fishing is an industry with substantial risk of injury, the trafficking survivors in our sample appear to have higher injury rates than a population of fishermen surveyed in Thai ports. In the International Labour Organization’s (ILO) survey of commercial fishermen in Thailand, 20.6% experienced injury, whereas 46.6% of fishermen in this study suffered injuries. Similarly, half of fishermen (53.8%) in our study were subjected to severe violence, whereas 10.1% of fishermen in the ILO survey were severely beaten [26].

Some of our findings contradict our original hypotheses. Men who received their wages had greater odds of injury; having documents does not appear to be protective for injuries or violence; and men with some language skills had greater odds of experiencing violence. Future work could explore whether the amount paid affects injury risk. Anecdotally, documents appear not to be protective against wider violations and abuse including police extortion and arrest [24,71,72]; documents may not be protective against poor working conditions and injuries either. Men who are fluent could talk back to managers or protest their conditions, which may anger and incur punishment from employers. Further research may help understand the ways that language skills might influence employment conditions and work relationships.

These patterns of abuse, occupational hazards, and injuries among some of the most exploited workers perhaps hint at the larger economic and structural forces that fuel, sustain, or neglect worker health and safety. Currently, in Southeast Asia, as in most other parts of the world, consumer demands, supply chains, and global manufacturing and trade are linked to poor or absent labour protections and extreme exploitation of workers who left home with aspirations of earning a fair wage [73,74]. Stronger enforcement of standard occupational health and safety measures is critical, including punishments for violations of occupational safety and health (OSH) regulations. Currently, in Southeast Asia OSH legislation is either non-existent or poorly enforced. Labour inspectorates have limited funding and yet pressure to expand their remits [75]. There is inconsistent and limited evidence on the effectiveness of labour inspections for injury prevention, with an urgent need for large-scale randomized trials to evaluate whether different types of inspection methods might reduce hazardous occupational exposures, illnesses and injuries [76]. Recent efforts have been made to regulate Thailand’s vast commercial fishing sector [77], but it remains to be seen whether these actions will also address workplace occupational hazards or provide workers with the health insurance schemes they need.

Informal sectors, such as agriculture, domestic work, and fishing, which are commonly comprised of mostly migrant workers, are vast and unregulated, and workers are often unaware of their legal rights—where these exist [78–80]. In Southeast Asia, under- developed legal systems, corruption problems and fear of authorities mean that for many migrants, regardless of status, legal recourse is uncommon and this reality is exploited by employers [24]. In many countries, migrant workers are prohibited from forming or leading trade unions and thus from collective bargaining to improve their working conditions [79]. However, there are examples of successful worker-driven efforts to negotiate better work conditions. The Migrant Worker Rights Network (MWRN) has been able to influence conditions in Thailand’s seafood export industry, for example, by supporting workers to negotiate with employers or filing complaints at government labour offices and education on labour law and rights. These types of assistance have led to positive outcomes in some cases, such as elimination of recruitment fees and workers being able to retain their identity documents [81].

Addressing sub-cultures of violence that have been normalized in some of these sectors may be more difficult. While there is growing evidence to inform prevention of violence against women and girls [82], efforts to reduce male on male interpersonal violence are not well-documented. We still know little about what works to reduce violence among men in the workplace and elsewhere [83]. Greater efforts need to be made by policy-makers and those driving global supply chains to address abuse of migrant workers, particularly those with insecure legal status.

Compensation is also a clear area for urgent improvement. Males who become permanently disabled from injuries face slim chances of future employment or ability to earn a living or support their families [84]. Failure to fulfill breadwinner expectations and feelings of guilt or shame post-trafficking is common among men [54,85]. Injuries have been also been associated with higher odds of incurring mental health disorders among labour-trafficked men [18]. Trafficked males who have incurred injuries will require both clinical services to treat physical wounds, and may also require mental health support and job placement services.

This study has some limitations. The study included users of post-trafficking services. As noted, screening processes and service eligibility for referral to post-trafficking services vary between countries and is often linked to differing legal definitions of trafficking. While we cannot generalize our findings to a larger population of trafficked males in the region [53], we believe our findings are indicative of a population of male post-trafficking service recipients affected by similar types of trafficking. Our study indicates the occupational hazards and abuses faced by men and boys who reach assistance after a trafficking experience. Findings on men's health needs will help service providers prioritize the care needs and make efficient use of often-scarce financial and human resources available to assist trafficked men. Furthermore, we anticipate that many of the hazards described in this study are similar to those faced by the larger population of men trafficked for fishing and manufacturing in the region.

Our analytic strategy (Directed Acyclic Graphs) did not permit estimation of effects of the primary exposures, hours worked per week and OHR score in the multivariable injuries analysis due to the presence of unobserved variables along the hypothesized backdoor paths from these exposures to injuries (see S1 Fig and S1 File), i.e. we have uncontrolled confounding in multivariable analyses for the primary exposures. Future research should include information about state implementation of OSH regulations and employer compliance, as well as worker attitudes towards OSH. Despite our use of DAGs, which theoretically guide causal estimation, our study is exploratory and hypotheses generating and did not aim to estimate causal effects of the exposures.

Given the widespread nature of human trafficking for labour and the enduring damage that these abuses cause to individuals, there is an urgent need to ensure survivors receive adequate post-trafficking care that responds to their health needs, as well as helps them to rebuild their lives and hopes for a better future. However, ultimately, the aim should be prevention. Without urgently implemented occupational health and safety and other interventions to confront these extreme forms of labour exploitation, individuals migrating for work will continue to endure pain and humiliation from simply seeking to earn a living for themselves and their families.

## Supporting Information

S1 Fig Complete list of Directed Acyclic Graphs used to inform covariate selection for multivariable models A–L presented in Table 4. (PDF)

S1 File Further information on use of Directed Acyclic Graphs (DAGs) to guide data analysis (PDF)
