# Supplementary material for: Labour Trafficking among Men and Boys in the Greater Mekong Subregion: Exploitation, Violence, Occupational Health Risks and Injuries
Source: PLoS One. 2016 Dec 16;11(12):e0168500. doi: 10.1371/journal.pone.0168500 (PMC5161368; doi:10.1371/journal.pone.0168500)

S1 Fig. Complete list of Directed Acyclic Graphs used to inform covariate selection for multivariable models A – L presented in Table 4

Fig 2. DAG for estimating effect of Hours on injuries (Model A)

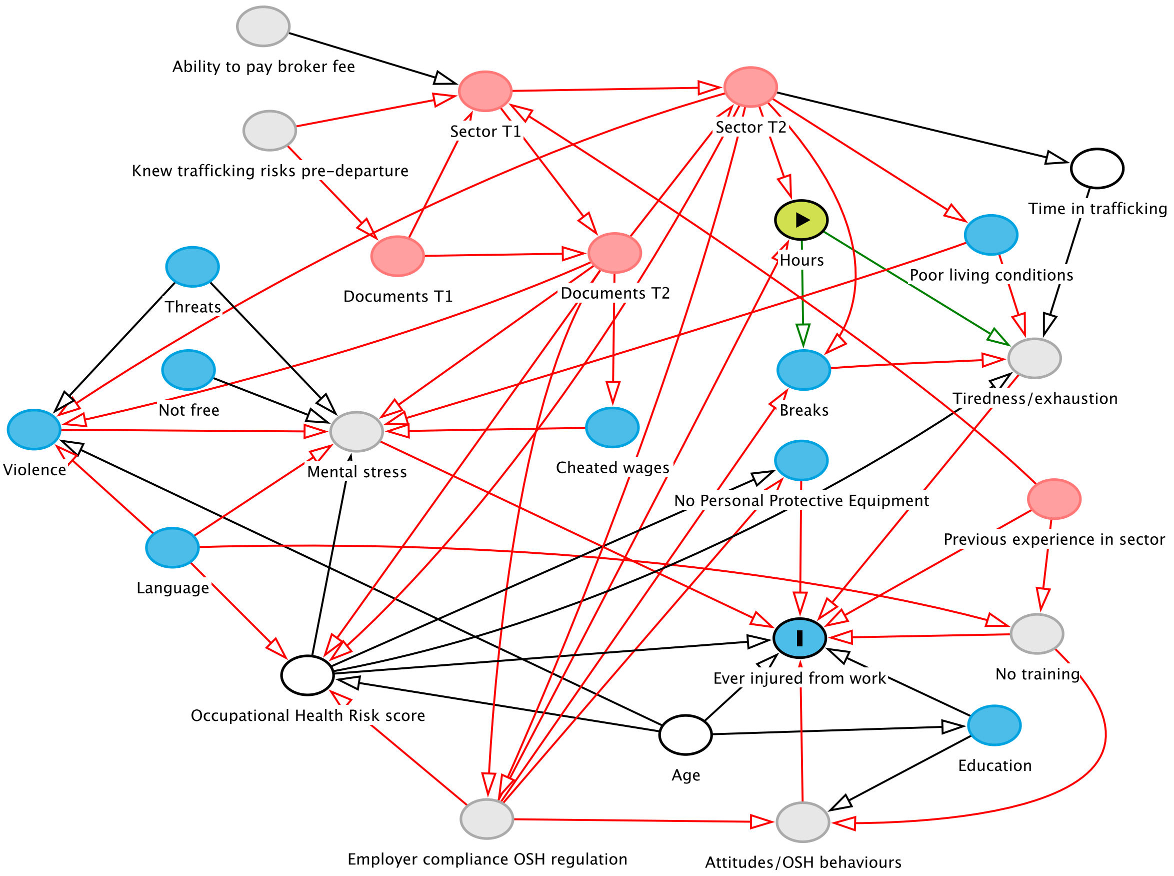

Fig 3. DAG for estimating effect of Occupational Health Risk (OHR) score on injuries (Model A)

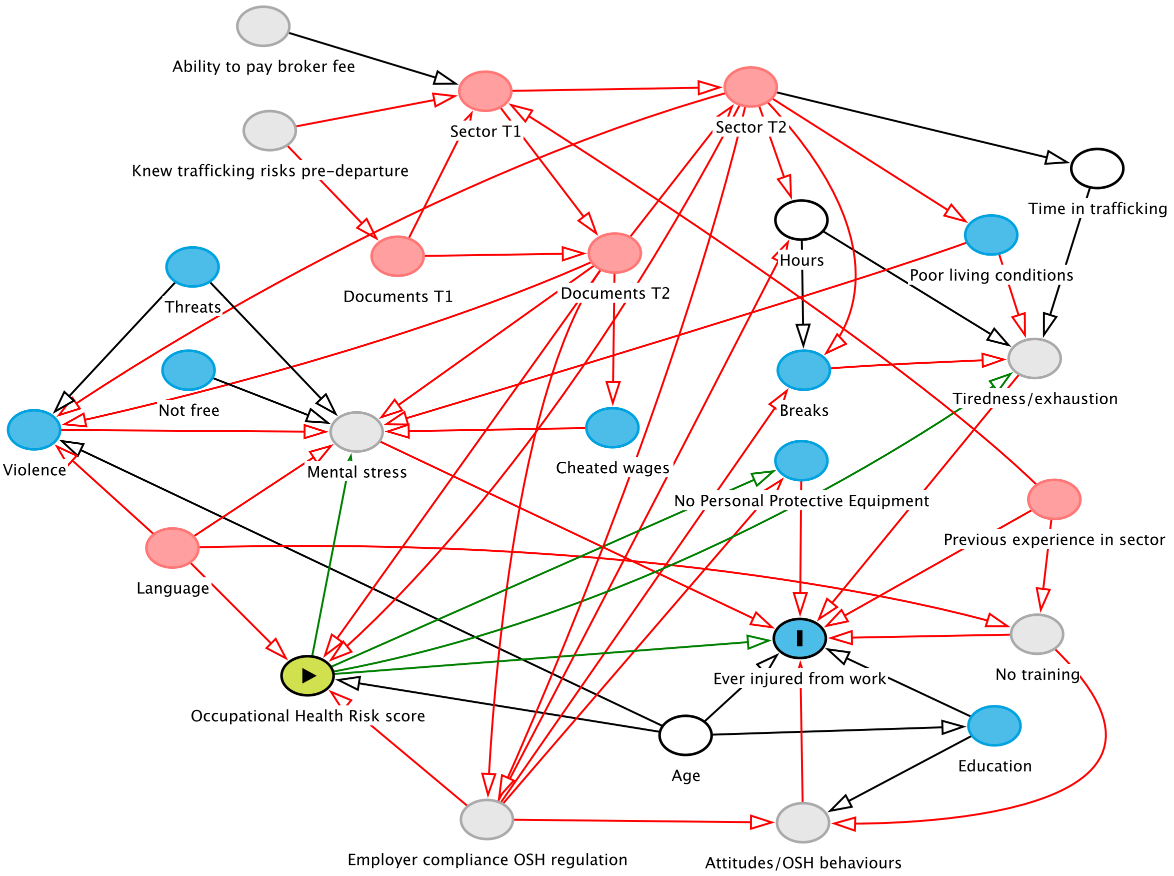

S1 Fig. Complete list of Directed Acyclic Graphs used to inform covariate selection for multivariable models A – L presented in Table 4

Fig 4. DAG for estimating effect of cheated wages on injuries (Model B)

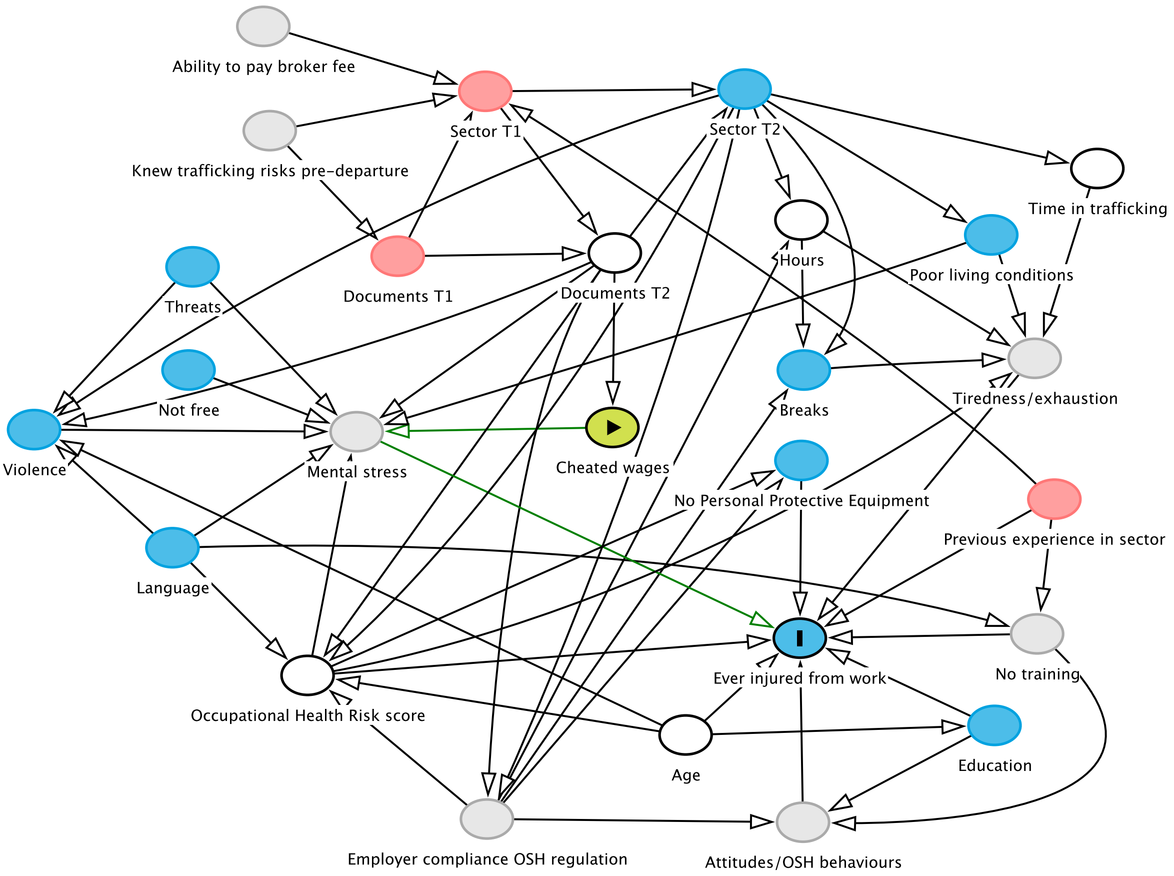

Fig 5. DAG for estimating effect of violence on injuries (Model C)

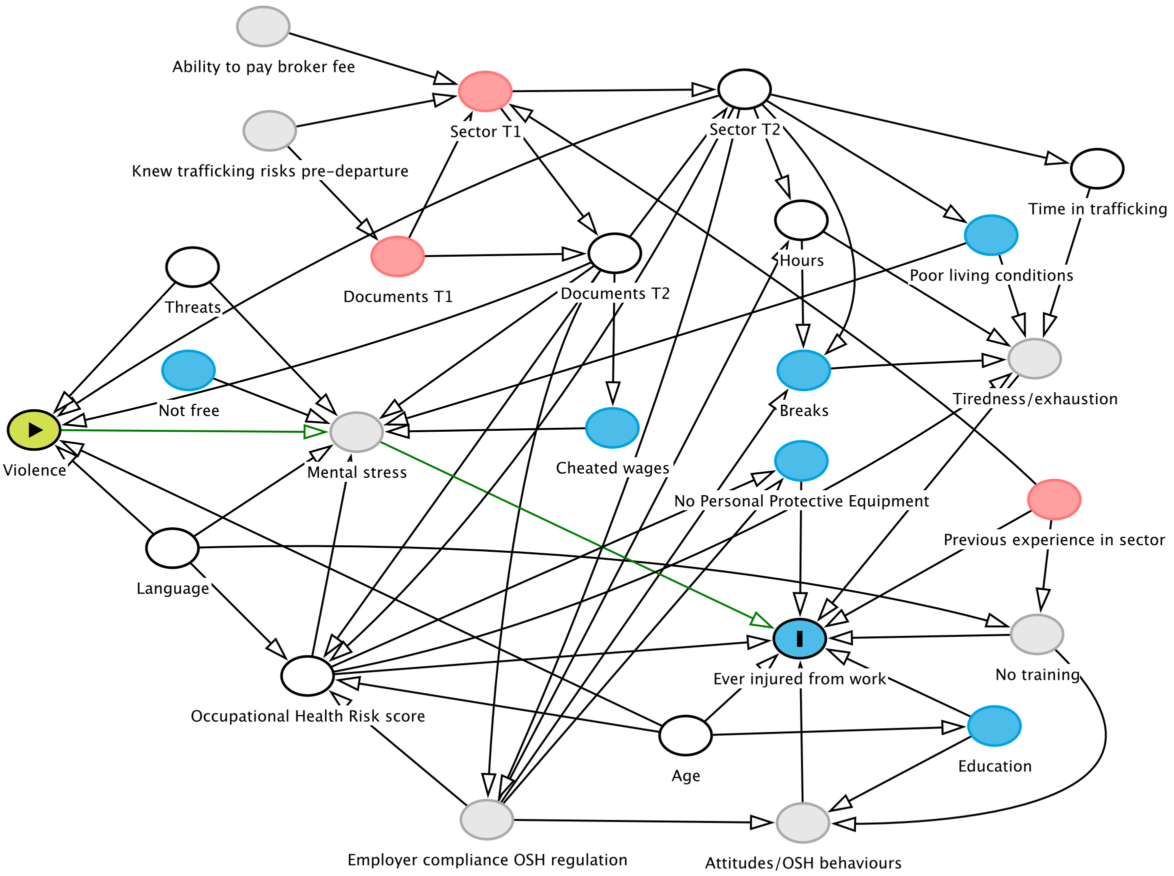

S1 Fig. Complete list of Directed Acyclic Graphs used to inform covariate selection for multivariable models A – L presented in Table 4

Fig 6. DAG for estimating effect of threats on injuries (Model D)

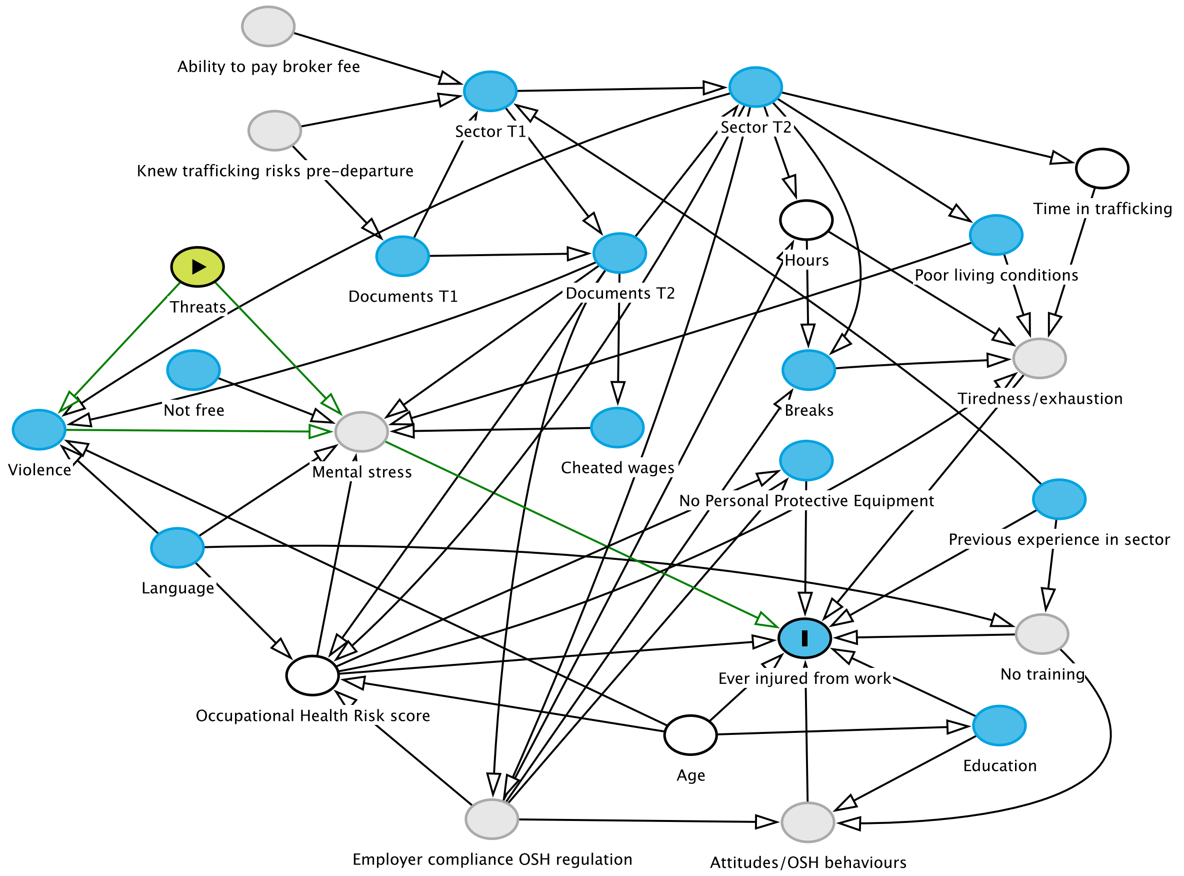

Fig 7. DAG for estimating effect of sector on injuries (Model E)

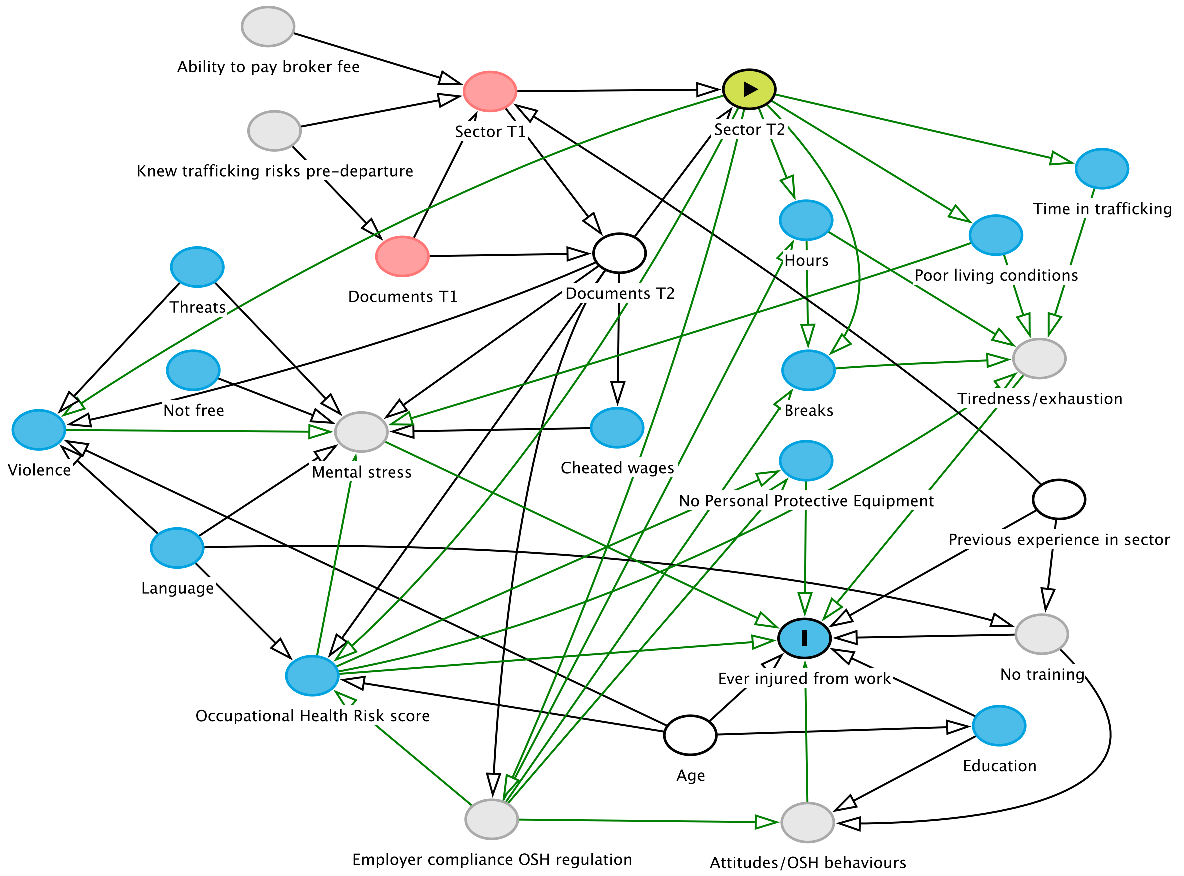

S1 Fig. Complete list of Directed Acyclic Graphs used to inform covariate selection for multivariable models A – L presented in Table 4

Fig 8. DAG for estimating effect of fluency on injuries (Model F)

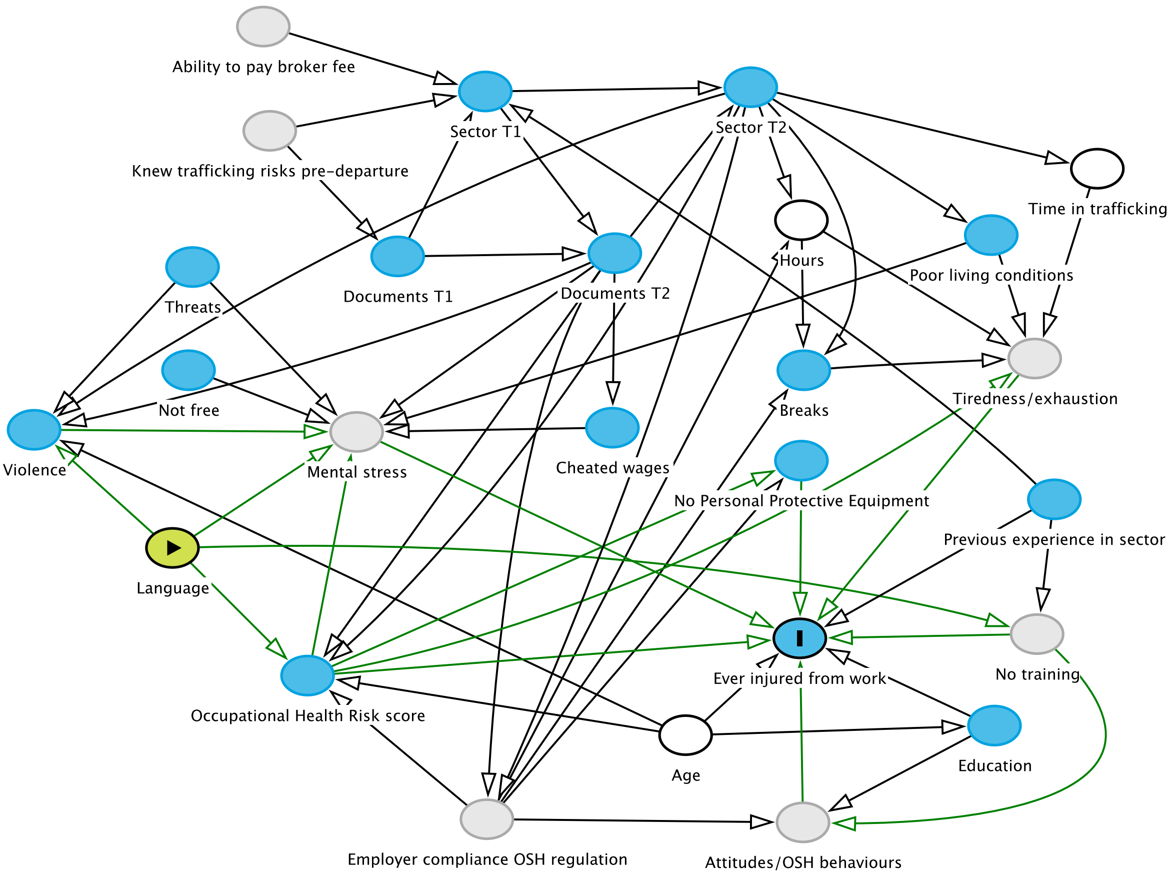

Fig 9. DAG for estimating effect of document possession on injuries (Model G)

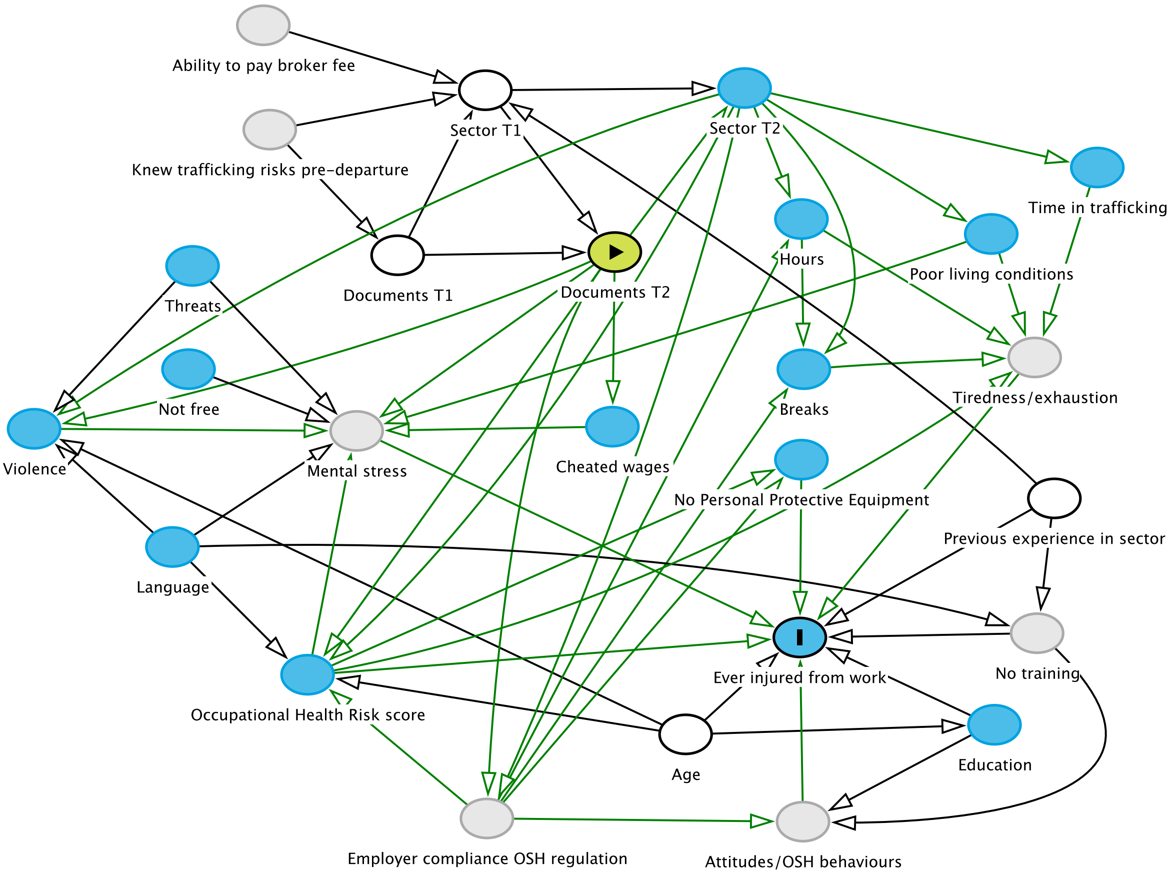

S1 Fig. Complete list of Directed Acyclic Graphs used to inform covariate selection for multivariable models A – L presented in Table 4

Fig 10. DAG for estimating effect of age on injuries (Model H)

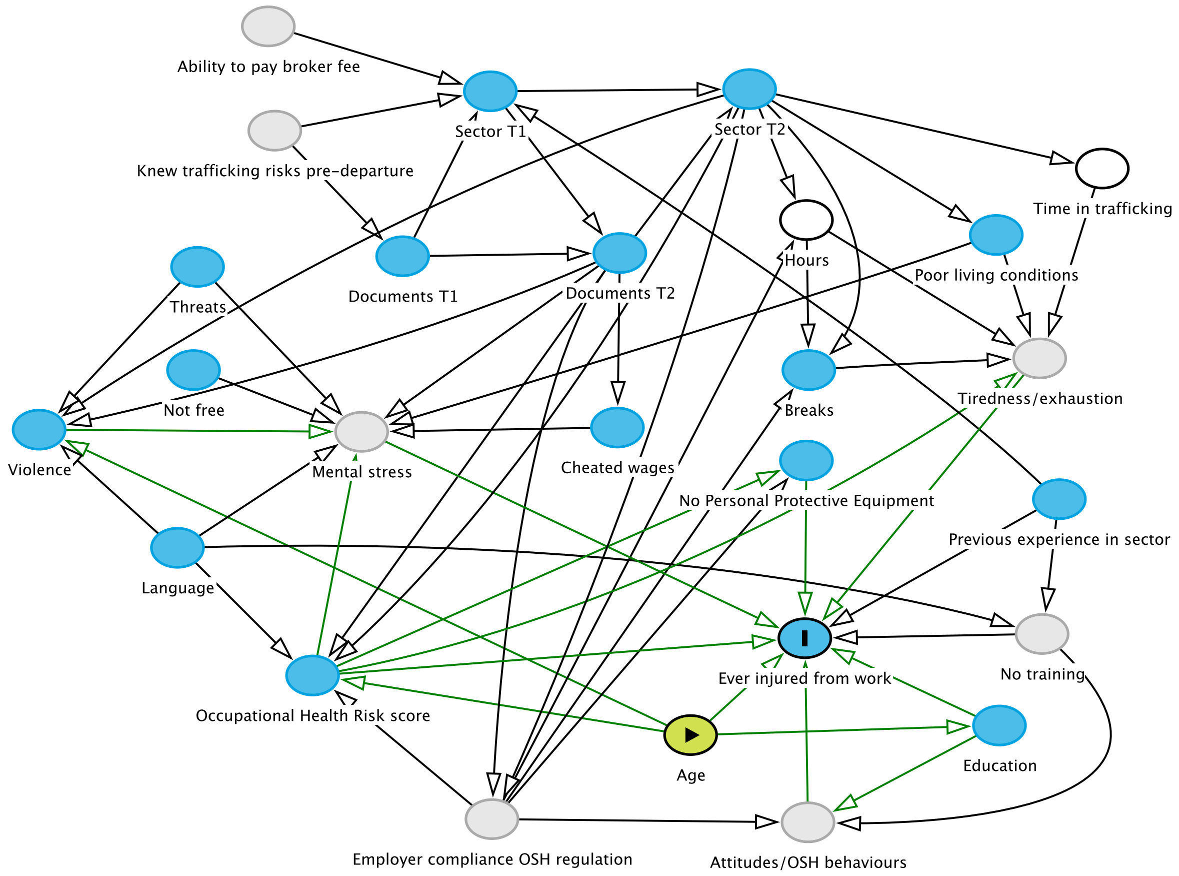

Fig 11. DAG for estimating effect of time in trafficking on injuries (Model I)

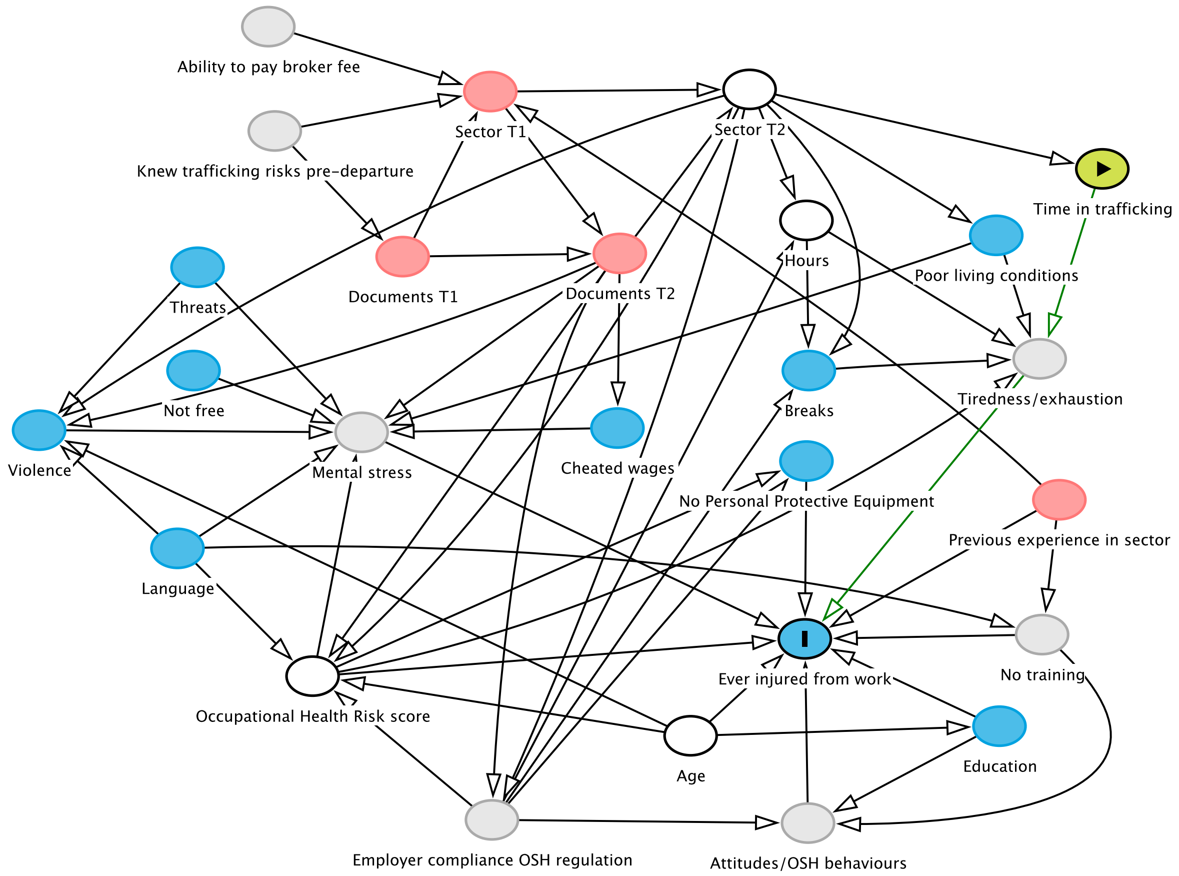

S1 Fig. Complete list of Directed Acyclic Graphs used to inform covariate selection for multivariable models A – L presented in Table 4

Fig 12. DAG for estimating effect of living conditions on injuries (Model J)

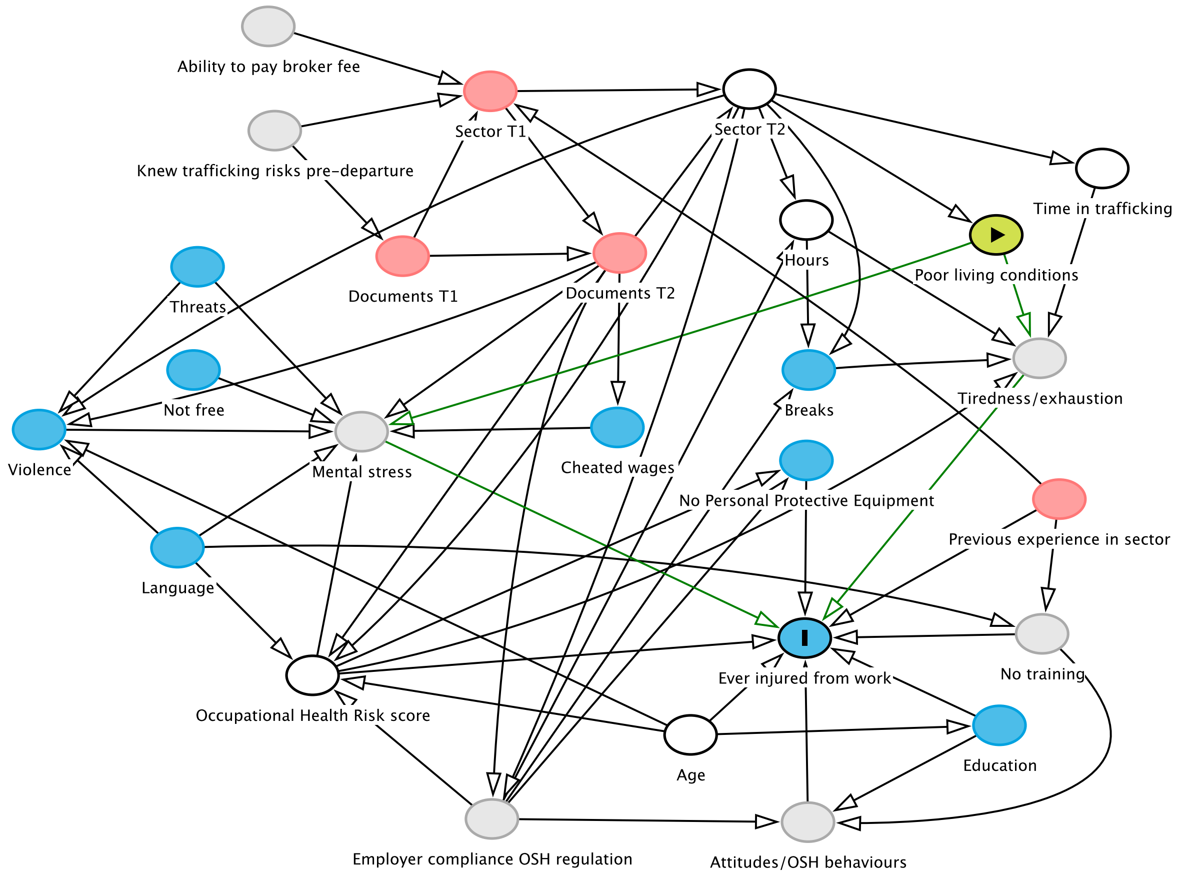

Fig 13. DAG for estimating effect of previous experience in sector on injuries (Model K)

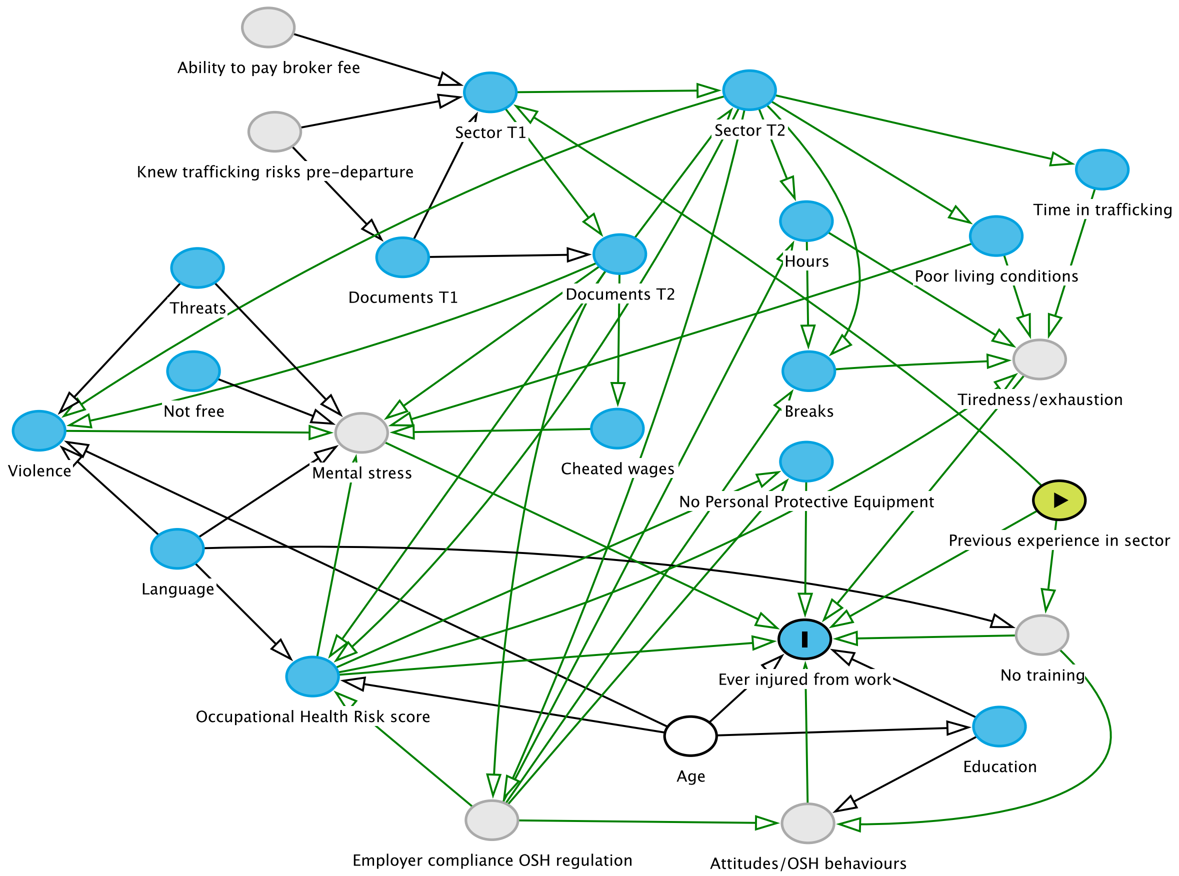

S1 Fig. Complete list of Directed Acyclic Graphs used to inform covariate selection for multivariable models A – L presented in Table 4

Fig 14. DAG for estimating effect of threats on violence (Model L)

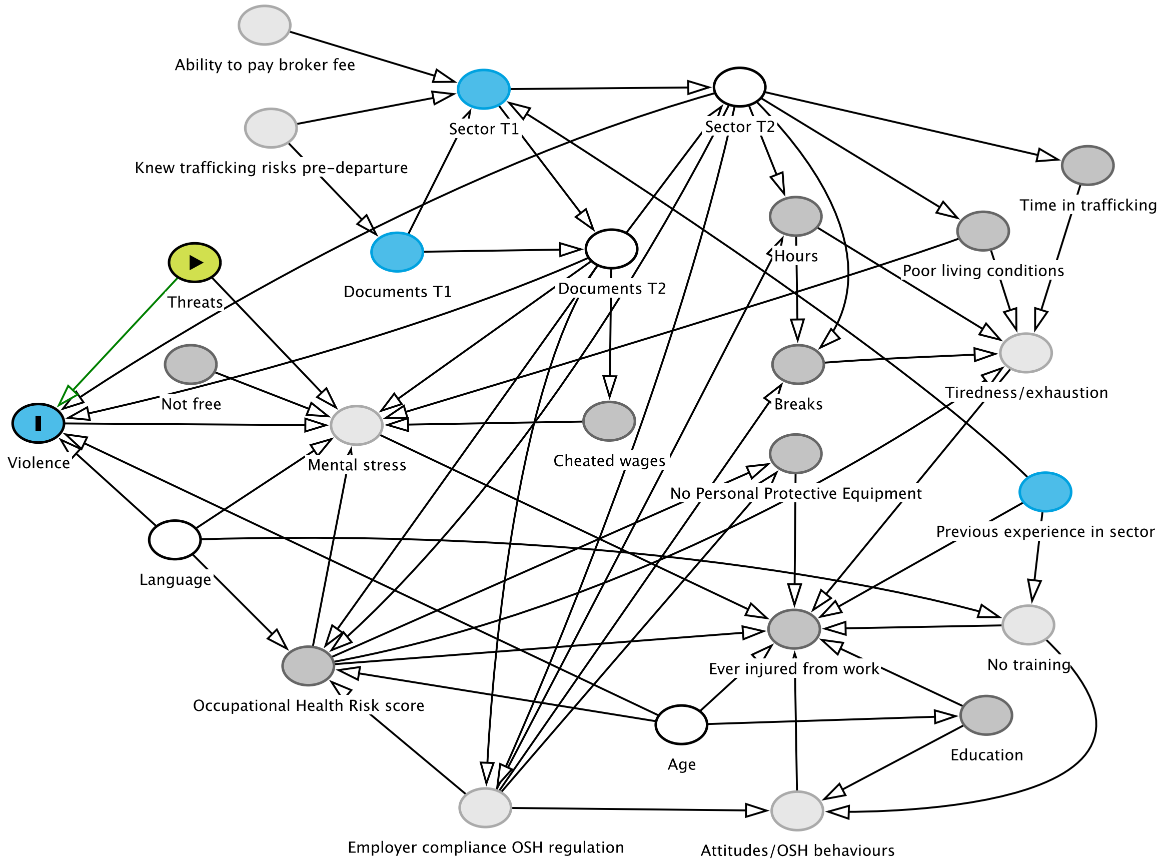

Fig 15. DAG for estimating effect of sector on violence (Model L)

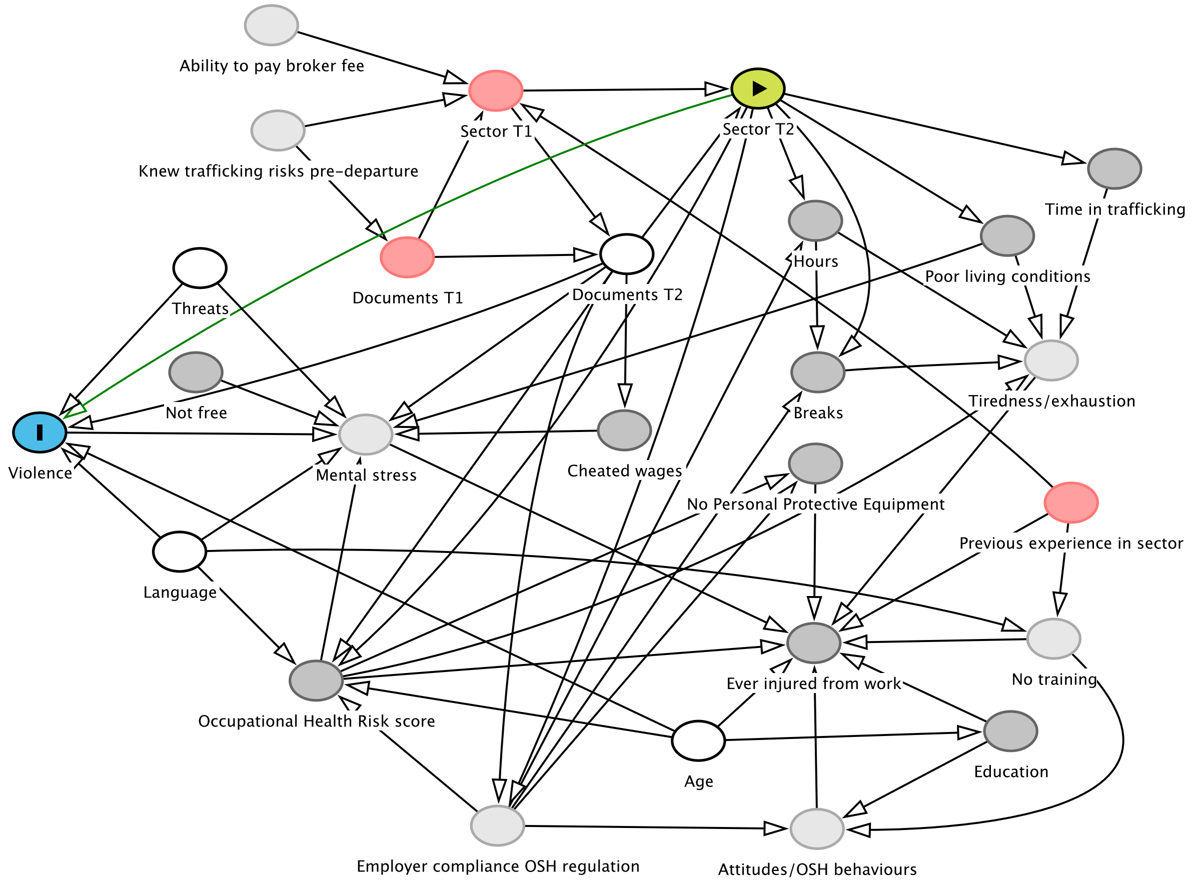

S1 Fig. Complete list of Directed Acyclic Graphs used to inform covariate selection for multivariable models A – L presented in Table 4

Fig 16. DAG for estimating effect of fluency on violence (Model L)

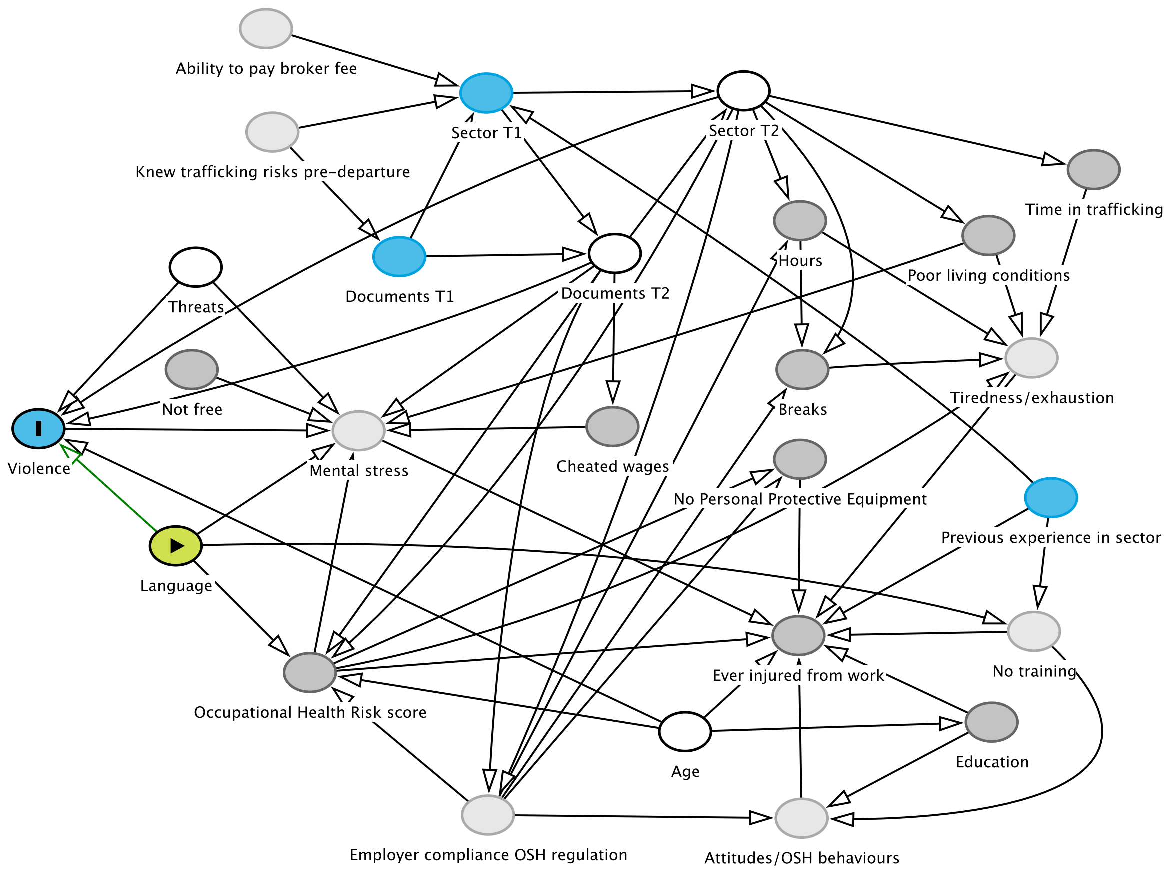

Fig 17. DAG for estimating effect of documents on violence (Model L)

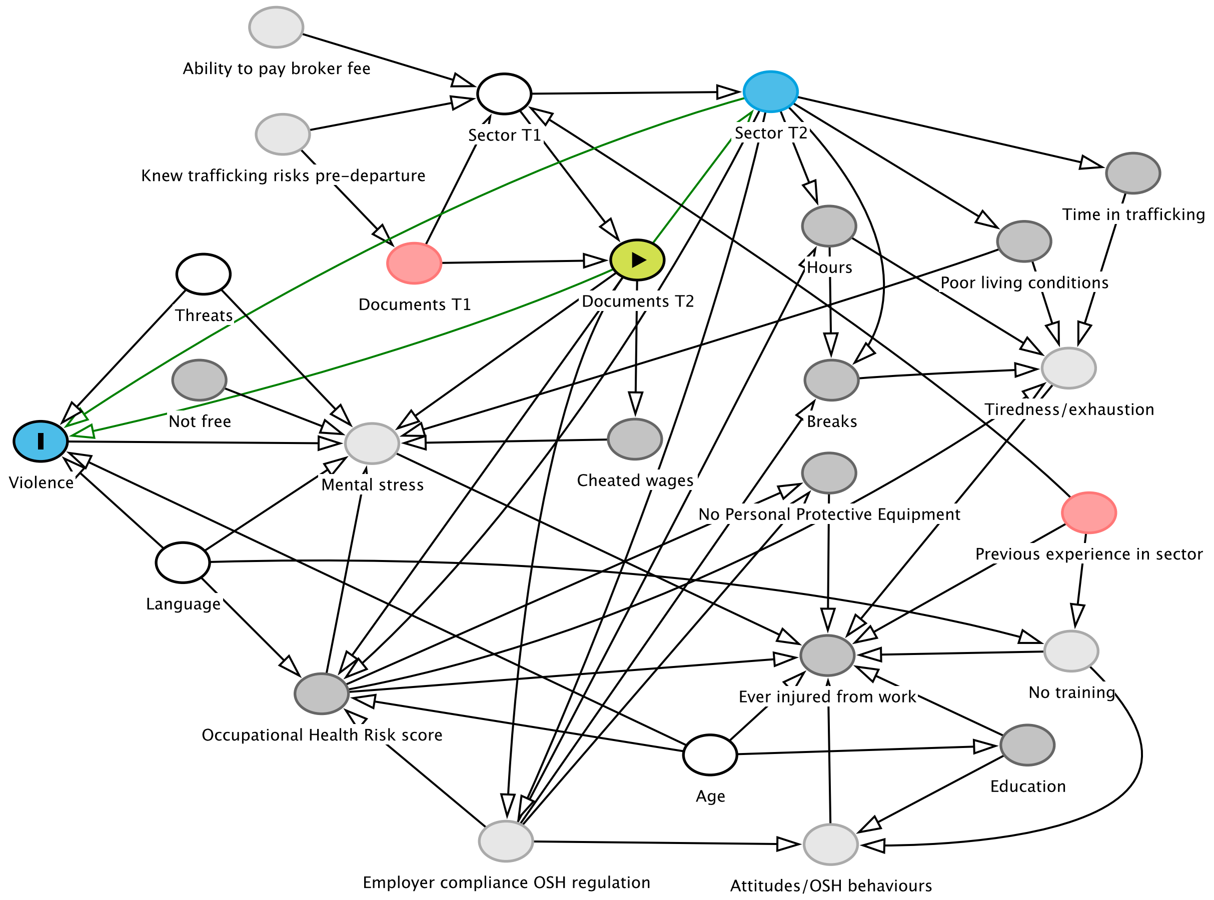

S1 Fig. Complete list of Directed Acyclic Graphs used to inform covariate selection for multivariable models A – L presented in Table 4

Fig 18. DAG for estimating effect of age on violence (Model L)

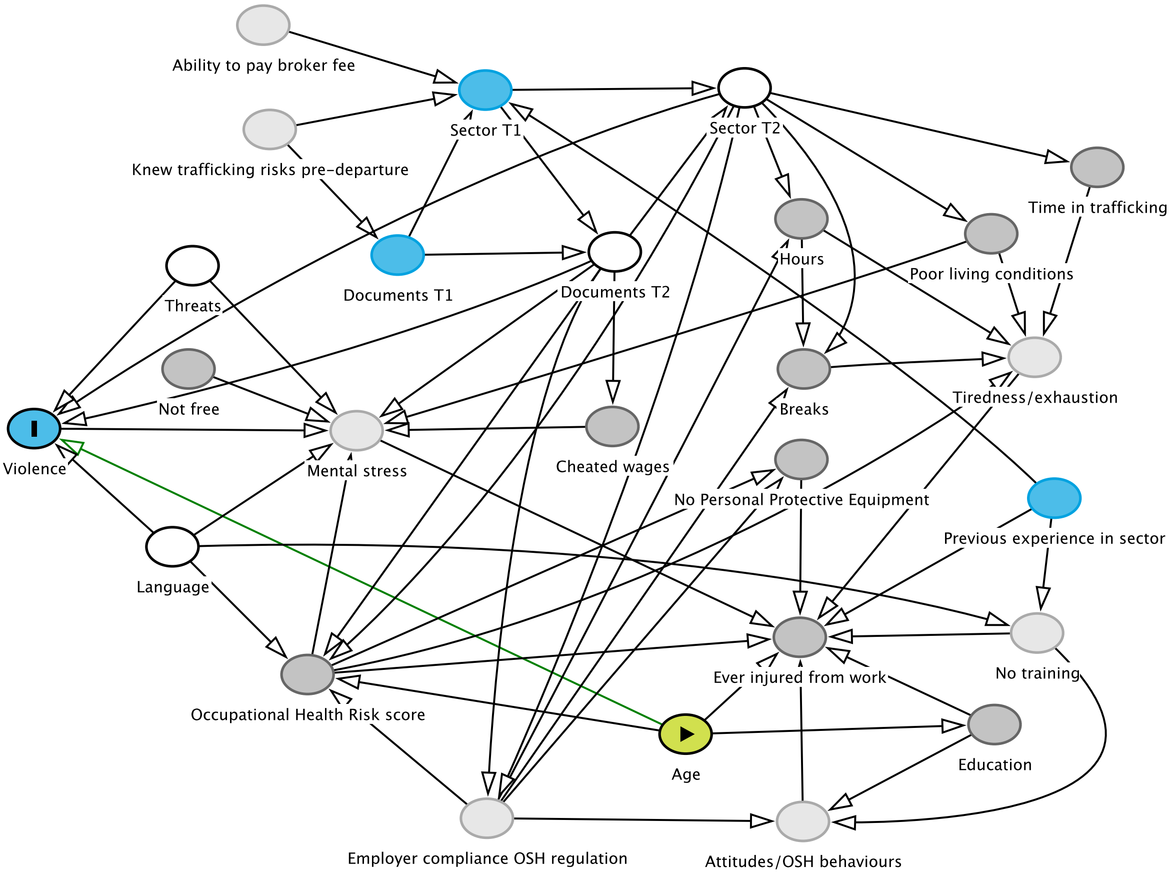

KEY

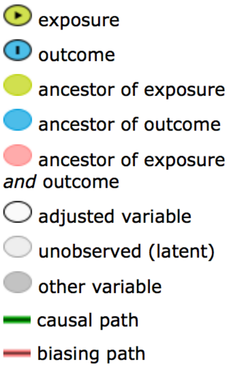

Supplement: S1 Fig — (PDF) [file pone.0168500.s001.pdf]
