# Supplementary material for: Labour Trafficking among Men and Boys in the Greater Mekong Subregion: Exploitation, Violence, Occupational Health Risks and Injuries
Source: PLoS One. 2016 Dec 16;11(12):e0168500. doi: 10.1371/journal.pone.0168500 (PMC5161368; doi:10.1371/journal.pone.0168500)
Supplement: S1 File — (PDF) [file pone.0168500.s002.pdf]

S1 File. Further information on use of Directed Acyclic Graphs (DAGs) to guide data analysis

Fig 1 illustrates our conceptual assumptions informing the statistical analysis. For example, we hypothesize that language proficiency affects mental stress (lack of fluency is stressful) and can also affect occupational risk score (lack of fluency to protest poor working conditions or understand instructions at work), which may subsequently increase injury risk (Fig 1). Variables hypothesized to affect violence included threats, language, documents, sector and age, with no particular hierarchy of proposed effects. DAGs were constructed using DAGitty software [1]. Whilst DAGs typically include one outcome per diagram, we include both outcomes (injuries and violence, marked "I") in Fig 1 for brevity (please see S1 Figs for complete list of DAGs). Blue variables are observed and grey are unobserved. Whilst the DAG shows that there may be other more proximal exposures (e.g. no protective equipment) that could affect injuries, our interest was in exploring the hypothesized relations for the variables mentioned above in addition to controlling for key factors such as months in trafficking and age. Sector and documents are represented temporally (although they are the same variable) as this permits the DAG to remain acyclic [2]. Possession of documents at T1 may influence which sector a migrant chooses to work in (self-selection), and at sector T1, the literature notes that fishermen may be more likely to have their documents withheld than males in other sectors [3].

The effects of the primary exposures, hours and OHR score, on injuries, could not be estimated because the variable "Employer compliance with OSH regulation" was unobserved and could not be controlled for to block off the back door paths between the exposure and outcome; please see S1 Fig, Figs 2 and 3, which show the resultant biasing paths between hours and OHR score to injuries. Back door paths in DAGs must be blocked to eliminate potential non-causal associations; the corollary in statistical criteria is that "Employer compliance with OSH regulation" is a confounder that can't be controlled for because it is unobserved [4]. Estimates for hours and OHR score in Table 4 should thus not be interpreted in any causal sense. However, hours and OHR score, alongside days worked/week, age and months in trafficking along with their polynomial terms, were controlled for in multivariable models for the secondary and tertiary exposures where this did not induce bias according to their respective DAGs (S1 Fig).

References

1. Textor J, Hardt J, Knüppel S. DAGitty: a graphical tool for analyzing causal diagrams. Epidemiol Camb Mass. 2011;22: 745. doi:10.1097/EDE.0b013e318225c2be

2. Greenland S, Pearl J, Robins JM. Causal diagrams for epidemiologic research. Epidemiol Camb Mass. 1999;10: 37–48.

3. Pearson E, Punpuing S, Jampaklay A, Kittisuksathit S, Prohmmo A. The Mekong Challenge: Underpaid, Overworked and Overlooked: The realities of young migrant workers in Thailand: Volume One. International Labour Organization (ILO) & the Institute for Social and Population Research (IPSR), Mahidol University; 2006.

4. Morgan SL, Winship C. Counterfactuals and Causal Inference: Methods and Principles for Social Research. 2 edition. New York, NY: Cambridge University Press; 2014.
